# Autophagy-dependent filopodial kinetics restrict synaptic partner choice during *Drosophila* brain wiring

**DOI:** 10.1038/s41467-020-14781-4

**Published:** 2020-03-12

**Authors:** Ferdi Ridvan Kiral, Gerit Arne Linneweber, Thomas Mathejczyk, Svilen Veselinov Georgiev, Mathias F. Wernet, Bassem A. Hassan, Max von Kleist, Peter Robin Hiesinger

**Affiliations:** 1 Division of Neurobiology, Institute for Biology, Freie Universität Berlin, 14195 Berlin, Germany; 2 Institut du Cerveau et de la Moelle Epinière (ICM) - Hôpital Pitié-Salpêtrière, Sorbonne Université, Inserm, CNRS, Paris, France; 3 MF1 Bioinformatics, Robert Koch-Institute, 13353 Berlin, Germany

**Keywords:** Cell biology, Developmental biology, Neuroscience

## Abstract

Brain wiring is remarkably precise, yet most neurons readily form synapses with incorrect partners when given the opportunity. Dynamic axon-dendritic positioning can restrict synaptogenic encounters, but the spatiotemporal interaction kinetics and their regulation remain essentially unknown inside developing brains. Here we show that the kinetics of axonal filopodia restrict synapse formation and partner choice for neurons that are not otherwise prevented from making incorrect synapses. Using 4D imaging in developing *Drosophila* brains, we show that filopodial kinetics are regulated by autophagy, a prevalent degradation mechanism whose role in brain development remains poorly understood. With surprising specificity, autophagosomes form in synaptogenic filopodia, followed by filopodial collapse. Altered autophagic degradation of synaptic building material quantitatively regulates synapse formation as shown by computational modeling and genetic experiments. Increased filopodial stability enables incorrect synaptic partnerships. Hence, filopodial autophagy restricts inappropriate partner choice through a process of kinetic exclusion that critically contributes to wiring specificity.

## Abstract (web summary)

The molecular mechanisms that restrict synapse formation with incorrect partners remain unclear. Here, authors use 4D imaging in developing Drosophila brains to show that filopodial kinetics are regulated by autophagy and this restricts inappropriate partner choice through a process of kinetic exclusion

## Introduction

Synapse formation and synaptic partner choice are based on cellular and molecular interactions of neurons in all animals^1–5^. Brain wiring diagrams are highly reproducible, yet most, if not all, neurons have the ability to form synapses with incorrect partners, including themselves^6,7^. During neural circuit development, spatiotemporal patterning restricts when and where neurons “see each other”^8–10^. Positional effects can thereby prevent incorrect partnerships, even when neurons are not otherwise prevented from forming synapses^7,11,12^. When and where neurons interact with each other to form synapses is a fundamentally dynamic process. Yet, the roles of neuronal interaction dynamics, e.g., the speed or stability of filopodial interactions, is almost completely unknown for dense brain regions in any organism. Our limited understanding of the dynamics of synaptogenic encounters reflects the difficulty to observe, live and in vivo, synapse formation at the level of filopodial dynamics in intact, normally developing brains^13,14^.

Fly photoreceptors (R cells) are the primary retinal output neurons that relay visual information with highly stereotypic synaptic connections in dense brain regions, namely the lamina and medulla neuropils of the optic lobe^15–17^. Intact fly brains can develop in culture, enabling live imaging at the high spatiotemporal resolution necessary to measure photoreceptor axon filopodial dynamics and synapse formation throughout the entire developmental period of circuit assembly^13,14,18^. Axonal filopodia inside the developing brain stabilize to form synapses through the accumulation of synaptic building material, but it remains unknown how limiting amounts of building material in filopodia are regulated^14^.

Macroautophagy (autophagy hereafter) is a ubiquitous endomembrane degradation mechanism implicated in neuronal maintenance and function^19^. Neuronal autophagy has been linked to neurodegeneration^20^ and synaptic function in the mature nervous system^21,22^. Comparably little is known about developmental autophagy in the brain. Functional neurons develop in the absence of autophagy^19,23,24^. In specific neurons in worms and flies, loss of autophagy leads to reduced synapse development^25,26^. By contrast, in the mouse brain, loss of autophagy in neurons leads to increased dendritic spine density due to defective pruning after synapse formation^27,28^. Despite numerous links to neurodevelopmental disorders, it remains unknown whether and how developmental autophagy can contribute to synaptic partner choice and circuit connectivity, especially in dense brain regions.

In this study, we show that loss of autophagy in *Drosophila* photoreceptor neurons leads to increased synapse formation and the recruitment of incorrect postsynaptic partners. Autophagy directly and selectively regulates the kinetics of synaptogenic axon filopodia, a phenotype that could only be revealed through live observation during intact brain development. Autophagic modulation of the kinetics of synaptogenic filopodia restricts what neurons “see each other” to form synapses, thereby critically contributing to the developmental program that ensures synaptic specificity during brain development.

## Results

We have previously observed the formation of autophagosomes at the axon terminals of developing photoreceptor neurons R1–R6 in the developing *Drosophila* brain, but their function has remained unknown^29^. Previous analyses of loss of autophagy in fly photoreceptors have not revealed any obvious developmental defects^24,30,31^.

### Autophagy affects neurotransmission and visual attention

To probe for previously undetected synaptic defects, we blocked autophagy in developing photoreceptor neurons using molecularly well-defined mutants for the essential autophagy proteins Atg7 and Atg6 (fly homolog of Beclin-1)^24,30^. We validated loss of the key autophagosome marker Atg8 in both *atg7* and *atg6* mutants (Supplementary Fig. 1a-b’, e). Rescue of *atg6* with the photoreceptor-specific driver GMR-Gal4 reversed this effect and led to a significant increase in Atg8-positive compartments compared with wild type (Supplementary Fig. 1c-c’, e).

**Fig. 1:**
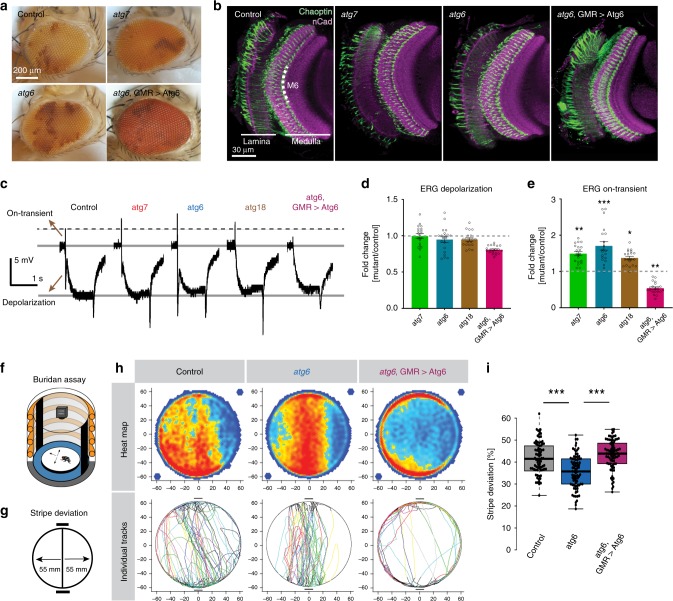
Autophagy deficiency in *Drosophila* photoreceptors leads to increased neurotransmission and visual attention. **a**, **b** Newly hatched (0-day-old) genetic mosaic flies with autophagy-deficient (*atg6* and *atg7* mutants) photoreceptors exhibit normal eye morphology (**a**) and axonal projections in the optic lobe (**b**). Repeated three times independently. **c** Representative electroretinogram (ERG) traces. Repeated three times independently. **d**, **e** Quantification of ERG depolarization (**d**) and on-transient (**e**) amplitudes relative to control. Rescue of *atg6* mutant photoreceptors with GMR > atg6 expression leads to overcompensation and increased autophagy (see Supplementary Fig. 1). *n* = 20 flies per condition. Two-tailed unpaired *t*-test with Welch’s correction; *p < 0.05, **p < 0.01, ***p < 0.001. Error bars denote mean ± SEM. **f** Buridan’s paradigm arena to measure object orientation response of adult flies, with two black stripes positioned opposite to each other as visual cues. **g** The parameter “stripe deviation”’ measures how much a fly deviates from a straight path between the black stripes in the arena. **h** Stripe fixation behavior of adult flies with *atg6* mutant photoreceptors, photoreceptors with upregulated autophagy (*atg6*, GMR > Atg6), and their genetically matched controls are shown on the population level (heatmap) and as individual tracks. Flies with *atg6* mutant photoreceptors show reduced stripe deviation, whereas increased autophagy (*atg6*, GMR > Atg6) leads to increased stripe deviation. **i** Quantification of stripe deviation. The error bars indicate the 25th percentile, the boxed area the 75th percentile, and the middle line of the boxplots indicates the median. *n* = 60 flies per condition, two-way ANOVA and Tukey’s HSD as post-hoc test; ***p < 0.001. Source data are provided as a Source Data file.

As expected, the eyes and axonal projections of photoreceptor neurons mutant for *atg6* or *atg7* in otherwise wild-type brains exhibited no obvious defects in fixed preparations (Fig. 1a, b). Photoreceptor neurons are known to exhibit neurodegeneration with aging^31^. To assay photoreceptor function directly following autophagy-deficient development, we therefore recorded electroretinograms (ERGs) from the eyes of newly eclosed flies. Autophagy-deficient photoreceptors exhibited normal depolarizing responses to light, indicating functional phototransduction and healthy neurons (Fig. 1c, d). Surprisingly, “on” transient amplitudes, which are indicative of synaptic transmission and the ability to elicit a postsynaptic response, were increased 30–50% in both mutants (Fig. 1c, e). Conversely, increased autophagy in transgenically rescued *atg6* photoreceptors reversed this effect and resulted in a significant reduction of “on” transients (Fig. 1c, e).

To further validate the effect of loss of autophagy on neurotransmission, we analyzed another autophagy mutant, *atg18*, which is recruited to the phagophore by PI3P (phosphatidylinositol 3-phosphate) and required for LC3 (Atg8) lipidation^32^. The *atg18*-null mutant behaved consistently as a hypomorph for autophagy. Loss of *atg18* in mutant clones reveals a significant, but (in contrast to *atg6* and *atg7*) not complete loss of Atg8-positive compartments (Supplementary Fig. 1d-d’, e). Similar to loss of *atg6* and *atg7*, loss of *atg18* in photoreceptors leads to increased neurotransmissions (Fig. 1c–e). We also performed RNA interference (RNAi) knockdown experiments for *atg5* and *atg16*^33,34^, validated decreased number of Atg8-positive compartments, and found similar increases in neurotransmission (Supplementary Fig. 2a–g).

Next, we asked whether loss of autophagy selectively in photoreceptors affected fly vision. We used the simple visual choice assay Buridan’s paradigm, in which wing-clipped flies walk freely in a circular, uniformly illuminated arena with two high-contrast black stripes placed opposite to each other (Fig. 1f)^35^. In this assay, flies with functional vision walk back and forth between the two high-contrast objects. We chose the parameter “stripe deviation,” which measures how much a single fly deviates from an imaginary line between two black stripes, as a behavioral read-out of visual attention (Fig. 1g). Flies with *atg6* or *atg7*-deficient photoreceptors were assayed and compared with their genetic background-matched controls. Surprisingly, in both mutants the flies with autophagy-deficient photoreceptors exhibited increased visual attention behavior (decreased stripe deviation) compared with their genetically matched controls (Fig. 1h, i and Supplementary Fig. 3). Increased autophagy in *atg6*-rescued photoreceptors reversed this effect again in an overcompensatory manner similar to ERG responses (Fig. 1h, i). This increase in autophagy also leads to an overcompensation of “center deviation,” i.e., how much a single fly moves away from the center (Supplementary Fig. 4a, b) but does not reduce the total distance walked (Supplementary Fig. 4c, d), leading to the observed increase in time spent walking the circumference of the arena (Fig. 1h). Visual attention is a higher-order behavior that requires functional basic vision. We therefore next tested basic motion vision using an optomotor assay with tethered, flying flies in a virtual flight arena^36,37^. Loss of autophagy in photoreceptors did not significantly affect the ability of flies to follow counter-clockwise and clockwise motion (see Methods; Supplementary Fig. 4e–l). We conclude that flies with photoreceptors that developed in the absence of autophagy can see, but their vision is characterized by both increased neurotransmission and increased visual attention.

### Autophagy-deficient photoreceptors form supernumerary synapses

**Fig. 2:**
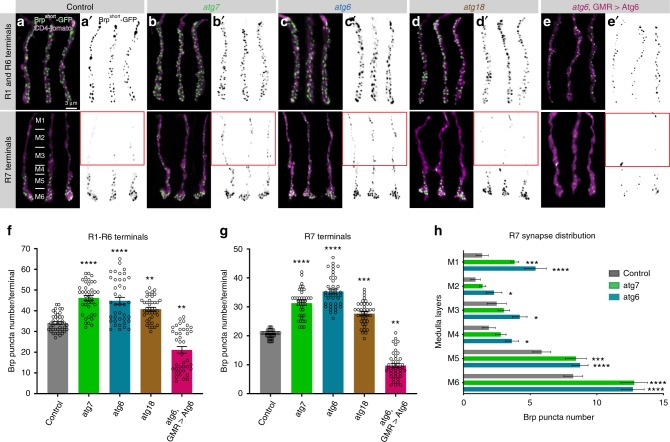
Autophagy-deficient *Drosophila* photoreceptors form supernumerary synapses. **a**–**e’** Representative images of R1–R6 and R7 photoreceptor axon terminals with Brp^short-^GFP marked active zones in wild-type (**a**, **a’**), *atg7* mutant (**b**, **b’**), *atg6* mutant (**c**, **c’**), *atg18* mutant (**d**, **d’**), and *atg6*, GMR > Atg6 (**e**, **e’**). R7 axon terminals are shown from distal (top) to proximal (bottom) medulla. Relative thicknesses of medulla layers are shown in Control R7 terminals panel (**a**) along R7 axon terminals. Red boxes show supernumerary synapses in loss of autophagy at distal part of R7 axon terminals. Repeated five to ten times independently with similar results. **f**, **g** Number of Brp puncta per terminal in R1-R6 (**f**) and R7 (**g**) photoreceptors. *n* = 40 terminals per condition. Kruskal–Wallis and Dunn’s as post-hoc test; **p < 0.01, ***p < 0.001, ****p < 0.0001. Error bars denote mean ± SEM. **h** Number of Brp puncta in distinct medulla layers along R7 axon terminals (see Methods for the definition of medulla layers and **a** for relative thicknesses of medulla layers). *n* = 22 terminals for control, *n* = 30 terminals for *atg7*, *n* = 27 terminals for *atg6*. Kruskal–Wallis and Dunn’s as post-hoc test; *p < 0.05, ***p < 0.001, ****p < 0.0001. Error bars denote mean ± SEM. Source data are provided as a Source Data file.

To assess whether the alterations in neurotransmission and vision were due to altered numbers of synapses, we generated sparse clones of photoreceptors R1–R6 and R7 expressing the active zone marker GFP-Brp^short^. This marker specifically localizes to presynaptic active zones without affecting synaptic development or function and is suitable for live imaging^14,38^. Loss of *atg6*, *atg7*, or *atg18*, as well as downregulation of *atg5* or *atg16* by RNAi resulted in a 25–80% increase in synapse numbers, whereas increased autophagy in rescued *atg6* mutant photoreceptors reversed this effect and significantly reduced synapse numbers (Fig. 2a–g and Supplementary Fig. 2h, i). In contrast, overexpression of *atg6* did not rescue *atg7* mutant photoreceptors, supporting the notion that *atg7* is absolutely required for autophagy and overexpression of *atg6* has no autophagy-independent effect in this system (Supplementary Fig. 5a, b).

Photoreceptors R1–R6 form columnar terminals in a single layer neuropil, whereas R7 axon terminals span six morphologically distinct layers and form the majority of synapses in the most proximal layer M6^17,39^. We were therefore surprised to see many supernumerary synapses in autophagy-deficient R7 axon terminals at more distal layers M1–M3 (Fig. 2h and red boxes in Fig. 2a–e’). These putative synapses along the distal shaft of autophagy-deficient R7 axons were stable based on live imaging of Brp^short^-labeled active zones with 15 min resolution over several hours at P70 (70% pupal development; Supplementary Movie 1). Brp stability is indicative of mature synapses and suggests that ectopic Brp puncta in fixed images are not the consequence of axonal transport defects or defective synaptic capture of Brp-positive transport vesicles. These observations raised the question whether loss of autophagy leads to genuine supernumerary synapses and, if so, whether these would be formed with correct postsynaptic partners.

### Autophagy-deficient R7s contact incorrect synaptic partners

**Fig. 3:**
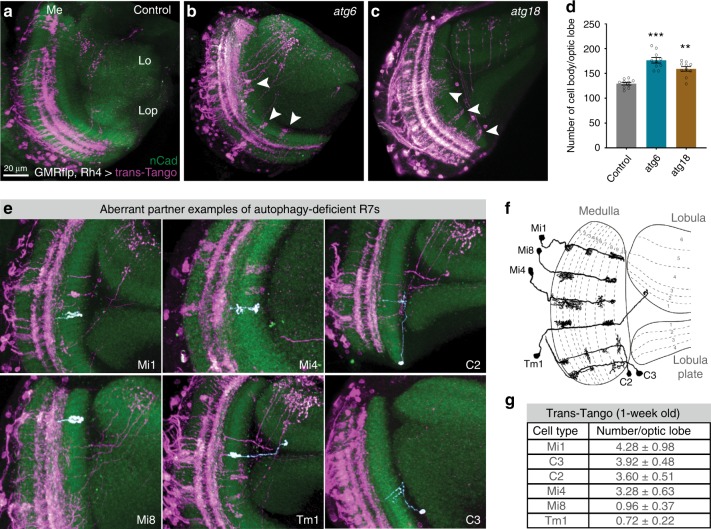
Loss of autophagy leads to synaptic connections with aberrant neuronal partners. **a**–**c** Neurons postsynaptic to control (**a**), *atg6* mutant (**b**), and *atg18* mutant (**c**) R7s are labeled with *trans*-Tango (see Methods for full genotypes; magenta = postsynaptic neurons, green = CadN, Me = medulla, Lo = lobula, Lop = Lobula plate). Arrowheads show postsynaptic neurons labeled for autophagy-deficient R7s but not for control R7s. Repeated three to five times independently with similar results. **d** Number of postsynaptic neurons per optic lobe for control, *atg6* mutant, and *atg18* mutant R7s based on *trans*-Tango-labeled cell body counts. *n* = 10 optic lobes per condition. One-way ANOVA and Tukey’s HSD as post-hoc test **p < 0.01, ***p < 0.001. Error bars denote mean ± SEM. **e** Examples of aberrant neuronal partners of autophagy-deficient R7s, with individual neurons pseudo-colored in white. **f** Schematic of dendritic and axonal arborization of aberrant neuronal partners (redrawn and adapted based on Golgi impregnations from Fischbach and Dittrich^46^). **g** Number of each aberrant neuronal partners per optic lobe from 1-week-old fly brains. Note that only ∼10% of R7s are mutant for *atg6* and *trans*-Tango labeling is dependent on synaptic strength between partners and progressively increase through age. See Methods for detailed *Drosophila* genotypes used to perform *trans*-Tango experiments. Source data are provided as a Source Data file.

The synaptic partners of R7 photoreceptors have been quantitatively characterized based on electron microscopy (EM) reconstruction of several medulla columns, revealing highly stereotypic connections^17^. The main postsynaptic target of R7 photoreceptors is the wide-field amacrine neuron Dm8^17,40^. Apart from Dm8s, R7s form fewer connections with Tm5 neuron subtypes that have dendritic fields spanning from M3 to M6^39,40^. To identify the postsynaptic partners of autophagy-deficient R7 photoreceptors, we used the recently developed anterograde *trans*-synaptic tracing method “*trans*-Tango,” which labels postsynaptic neurons for a given neuron without a need for previous knowledge about the nature of the connections^41^. In brief, the method is based on a synthetic signaling pathway that is introduced into all neurons in the animal, but only *trans*-synaptically activated by a tethered ligand expressed in a specific presynaptic neuron^41^. We used an R7-specific driver (Rhodopsin4-Gal4) and restricted its expression to mutant R7 photoreceptors, whereas all other neurons, including all postsynaptic partners, are wild type. Consistent with known postsynaptic targets of R7s, *trans*-Tango with wild-type R7s mainly labeled Dm8s and Tm5s (Fig. 3a and Supplementary Fig. 6). By contrast, loss of *atg6* or *atg18* in R7s led to a more widespread labeling of postsynaptic neurons (Fig. 3b, c) and an overall increase of the number of postsynaptically connected cells, as expected for supernumerary functional synapses (Fig. 3d). Through application of a sparse-labeling protocol of *trans*-Tango, we further identified several cell types, including Mi1, Mi4, Mi8, Tm1, C2, and C3, which are not normally postsynaptic to R7 based on connectome data^15,17,42,43^ (Fig. 3e, f). Mi1 and Mi4, e.g., are part of the motion-detection pathway, to which R7 is not known to provide input^44,45^. Notably, the number of individual neurons detected for these six ectopically connected neurons correlated distinctly with the position of their presumptive dendritic trees: Mi1, C3, and C2 were most often labeled and all three have presumptive dendrites in layers M1 and M5 (Fig. 3f, g)^46^; most ectopic R7 synapses were detected in layer M1, M5, and M6 (Fig. 2h); at the other end of the spectrum, Mi8 and Tm1 were both four-to fivefold less often detected and have presumptive dendrites in layer M2 and M3, where we counted fewer ectopic synapses (Figs. 2h and 3f, g)^46^. These findings suggest that the postsynaptic neurons labeled by *trans*-Tango are incorrect partners connected through axon-dendritic contacts with R7.

### Synapses with incorrect postsynaptic neurons are functional

**Fig. 4:**
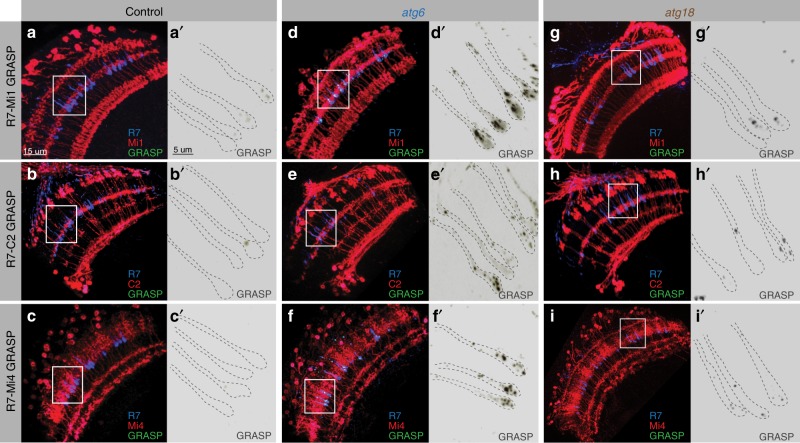
Synaptic connections between autophagy-deficient R7s and aberrant postsynaptic partners are functional based on activity-dependent GRASP. **a**–**c’** Activity-dependent GRASP between control R7s and Mi1s (**a**, **a’**), C2s (**b**, **b’**), and Mi4s (**c**, **c**’) show that wild-type R7s very rarely form synaptic connections, if any, with Mi1, C2, and Mi4 neurons. **d**–**f’** Activity-dependent GRASP between *atg6* mutant R7s and Mi1s (**d**, **d’**), C2s (**e**, **e’**), and Mi4s (**f**, **f’**) show widespread active synaptic connections between autophagy-deficient R7s and aberrant postsynaptic partners. **g**, **i’**, Activity-dependent GRASP between *atg18* mutant R7s and Mi1s (**g**, **g’**), C2s (**h**, **h’**), and Mi4s (**i**, **i’**) show less frequent active synaptic connections compared with *atg6* mutants. Note that Atg18 loss-of-function does not block autophagosome formation as effective as Atg6 loss-of-function (see Supplementary Fig. 1). Regions inside yellow rectangles are shown in close-up images as single greyscale GRASP channels. See Methods for Mi1, Mi4, and C2-specific LexA drivers and detailed *Drosophila* genotypes used to perform GRASP experiments. Repeated three times independently with similar results.

To test whether these contacts are functional synapses, we next used the activity-dependent GRASP method (Green fluorescent protein [GFP] reconstitution across synaptic partners), which is based on *trans*-synaptic complementation of split GFP only when synaptic vesicle release occurs^47,48^. Based on available cell-specific driver lines and the underlying genetics, we could test three of the ectopic pairs identified with *trans*-Tango: potential synapses between R7 and Mi1, C2 or Mi4. For all three cases, wild-type neurons rarely showed isolated synaptic signals (Fig. 4a–c’). In contrast, *atg6* mutant photoreceptors formed abundant synapses in all three cases (Fig. 4d–f’). All three incorrect synaptic pairings were validated for *atg18* mutant photoreceptors, albeit at lower levels (Fig. 4g–i). These findings based on activity-dependent GRASP also indicate that the *trans*-Tango results were not due to an effect of altered autophagy on the ectopically expressed proteins of the *trans*-Tango system. We conclude that loss of autophagy in R7 photoreceptor terminals leads to ectopic synapse formation with inappropriate postsynaptic neurons.

Taken together, our observations reveal that loss of autophagy in photoreceptors does not affect overall axon terminal morphology and transmission of visual input, but selectively leads to increased synapse formation, which includes inappropriate postsynaptic partners, and increased visual attention behavior. However, how does defective autophagy at the developing presynapse affect synaptic partner choice mechanistically?

### Autophagy modulates the stability of synaptogenic filopodia

**Fig. 5:**
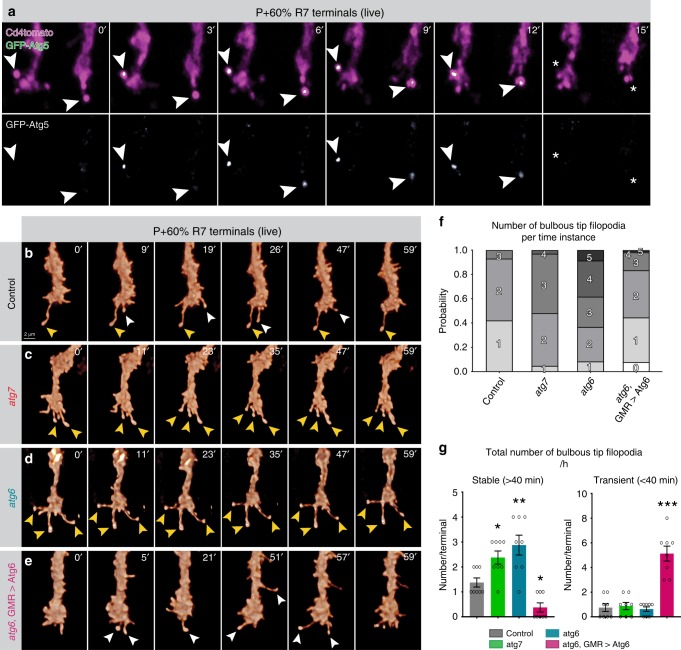
Autophagy regulates the stability of synaptogenic filopodia at axon terminals. **a** Live imaging of GFP-Atg5-expressing R7 axon terminals in intact, developing *Drosophila* brain shows formation of autophagosomes at the bulbous tips of synaptogenic filopodia^14^ followed by the collapse of filopodia (P + 60%). Repeated three times independently with similar results. **b**–**e** Live imaging of R7 axon terminals at P + 60% (during synaptogenesis) revealed increased stability of synaptogenic filopodia in autophagy-deficient R7 terminals (**c**, **d**) and decreased stability in R7 terminals with upregulated autophagy (**e**) compared with control (**b**). Yellow arrowheads: stable synaptogenic filopodia; white arrowheads: unstable bulbous tip filopodia. Repeated five to ten times independently with similar results. **f** Number of concurrently existing bulbous tip filopodia per R7 axon terminal per time instance. **g** Total number of synaptogenic filopodia per R7 axon terminal per hour. Autophagy-deficient R7 terminals exhibit significantly more stable synaptogenic filopodia (>40 min), whereas upregulated autophagy leads to filopodia destabilization. *n* = 8 terminals per condition. One-way ANOVA and Tukey’s HSD as post-hoc test; *p < 0.05, **p < 0.01, ***p < 0.001. Error bars denote mean ± SEM. Source data are provided as a Source Data file.

To test when and where exactly autophagosomes function during synapse formation, we performed live-imaging experiments of autophagosome formation in developing R7 axon terminals in developing brains. Autophagosomes have previously been shown to form at axon terminals in vertebrate primary neuronal cell culture using the temporal series of autophagosome progression reporters GFP-Atg5 (early) and GFP-Atg8a (late)^49^. We used the same markers to track autophagosome progression after validation that overexpression of neither of these proteins affect development, neurotransmission, or synapse numbers in fly photoreceptors (Supplementary Fig. 7). Surprisingly, we detected autophagosome formation based on these probes selectively at the rare, bulbous tips of synaptogenic filopodia of R7 axon terminals, followed by filopodial collapse (Fig. 5a, Supplementary Fig. 8, and Supplementary Movie 2).

We have recently shown that altered numbers of synaptogenic filopodia lead to changes in synapse numbers^14^. We therefore tested the effects of a loss of autophagy on R7 axon terminal filopodial dynamics during synapse formation (developmental time point P60). Both *atg6* and *atg7* mutants exhibited selectively increased lifetimes of the population of long-lived axonal filopodia compared with wild-type and *atg6*-rescued photoreceptors (Supplementary Fig. 9 and Supplementary Table 1). Wild-type axon terminals only formed one to two synaptogenic filopodia, as characterized by their bulbous tips, at any point in time (Fig. 5b, f–g), which previously led us to propose a serial synapse formation process that slowly spreads out the formation of 20–25 synapses over 50 h^14^ (also see Supplementary Movie 3). In contrast, loss of *atg6* or *atg7* in R7 axon terminals led to three to four synaptogenic filopodia at any time point (Fig. 5c, d, f, g and Supplementary Movie 3). As expected for synaptogenic filopodia, almost all supernumerary bulbous tips were stable for more than 40 min (Fig. 5g). Increased autophagy in *atg6*-rescued mutant photoreceptors reversed this effect and lead to a significant reduction and destabilization of synaptogenic filopodia (Fig. 5e–g and Supplementary Movie 3). By contrast, *atg6* overexpression in *atg7* mutant photoreceptors did not alter the increased filopodial stability of *atg7* mutants (Supplementary Fig. 5c), indicating that levels of *atg6* affect filopodia stability in an autophagy-dependent manner. Consistent with selective autophagosome formation in synaptogenic filopodia tips, the changes to filopodial dynamics were remarkably specific to long-lived, synaptogenic filopodia (Fig. 5b–g, Supplementary Fig. 9, and Supplementary Table 1). In sum, analyses of R7 axon terminal dynamics during synapse formation in the intact brain revealed autophagosome formation in synaptogenic filopodia and a specific effect of autophagy function on the kinetics and stability of these filopodia.

### A filopodial dynamics model predicts altered synapse numbers

**Fig. 6:**
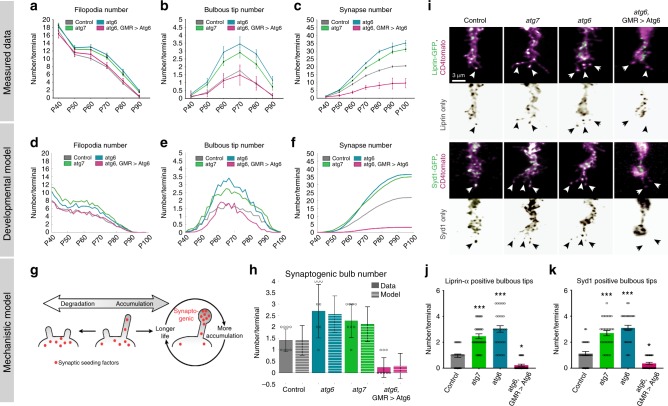
Loss of autophagy increases the number of synaptogenic filopodia through defective synaptic seeding factor degradation, leading to increased synapse formation throughout development. **a**–**c** Quantification of filopodia numbers (**a**), synaptogenic filopodia numbers (**b**), and Brp puncta numbers (**c**) during synaptogenesis (P40–P90) per R7 axon terminal based on fixed data. *n* = 40 terminals per condition. **d**–**f** Markov State Model simulation based on data in (**a**) and live data at P + 60% (Fig. 5) for filopodia numbers (**d**), synaptogenic filopodia numbers (**e**), and Brp puncta numbers per R7 axon terminal (**f**). **g** The mechanistic model: accumulation of synaptic seeding factors stabilizes synaptogenic filopodia; autophagic degradation of synaptic seeding factors destabilizes filopodia. **h** Measured (solid bars) and simulated (striped bars) synaptogenic filopodia numbers at P + 60% (the simulated data are based on synaptic seeding factor availability, see Supplementary Fig. 6). *n* = 8 axon terminals from independent live-imaging sessions. **i** Representative images of synaptic seeding factors (Syd-1 and Liprin-α) localizing to synaptogenic filopodia. Repeated three times independently with similar results. **j**, **k** Quantifications of the number of Liprin-α (**j**) and Syd-1 (**k**) positive synaptogenic filopodia. *n* = 30 terminals per condition. Kruskal–Wallis and Dunn’s as post-hoc test; *p < 0.05, ***p < 0.001. Error bars denote mean ± SEM. Source data are provided as a Source Data file.

Next we asked whether the observed changes to the kinetics of synaptogenic filopodia are sufficient to quantitatively explain changes in synapse formation throughout the second half of fly brain development. We first counted the numbers of overall filopodia, bulbous tip filopodia, and synapses at time points every 10 h between P40 and P100 in fixed preparations (Fig. 6a–c). Compared with control, loss of *atg6* or *atg7* in photoreceptors led to mild increases in overall filopodia, while leaving the rates of change largely unaltered between time points (Fig. 6a). In contrast, numbers of synaptogenic bulbous tip filopodia are increased twofold throughout the main period of synapse formation (P60–P80; Fig. 6b and Supplementary Fig. 10). Synapse numbers, based on presynaptic *Brp*^*short*^ labeling, commences indistinguishably from wild type, but then increases at a higher rate throughout brain development (Fig. 6c).

We previously developed a data-driven Markov state model that predicts the slow, serial development of synapses throughout the second half of brain development based on stochastic filopodial exploration and one-by-one selection of synaptogenic filopodia^14^. To test how autophagy-dependent changes of filopodial kinetics affect synapse formation in the model, we used the measured live dynamics of filopodia at P60 (Fig. 5b–g, Supplementary Fig. 9, and Supplementary Tables 1–3) together with the measured fixed time points data for filopodia (Fig. 6a, b and Supplementary Fig. 10) as input. As shown in Fig. 6d–f, the model recapitulates all aspects of synaptogenic filopodial dynamics and synapse formation for both loss and upregulation of autophagy. The model thereby shows that the measured changes in filopodial kinetics, and specifically altered stabilization of synaptogenic filopodia, are sufficient to cause the observed alterations in synapse formation over time (see “Mathematical modeling” in Methods). These findings raise the question how autophagy can specifically regulate the kinetics of synaptogenic filopodia mechanistically.

### Degradiation of synaptic proteins tunes filopodia kinetics

We have previously shown that the early synaptic seeding factors Syd-1 and Liprin-α are allocated to only one to two filopodia at any given time point, and that their loss leads to the destabilization of synaptogenic filopodia and a loss of synapses^14^. Autophagy is a protein degradation pathway that affects filopodia stability in opposite ways in loss- vs. gain-of-function experiments. We therefore hypothesized that autophagic degradation may directly regulate the availability of synaptic building material in filopodia. We first tested this idea using a second Markov state model that simulates the stabilization of filopodia as a function of seeding factor accumulation and degradation on short time scales (Fig. 6g and Supplementary Fig. 11a). In this “winner-takes-all” model, synaptic seeding factors are a limiting resources in filopodia that increase filopodia lifetime, which in turn increases the time available for further accumulation of synaptic seeding factors, creating a positive feedback loop^14^. If autophagy plays a role in the degradation of synaptic seeding factors, then decreased autophagic degradation of synaptic seeding factors should lead to more synaptogenic filopodia, whereas increased autophagic degradation should reduce synaptogenic filopodia through further restriction of the limiting resource (Fig. 6g and Supplementary Fig. 11a). The simulations show that the measured number of synaptogenic filopodia (Fig. 6h) and their lifetimes (Supplementary Fig. 11) can be quantitatively explained by degradation and thus availability of synaptic seeding factors for both loss and upregulation of autophagy at P60. Specifically, the number of long-lived filopodia at autophagy-deficient axon terminals was increased compared with control and conversely increased autophagic activity led to a decreased lifespan of filopodia as measured (Supplementary Fig. 9 and Supplementary Table 1). Hence, the mechanistic model predicts that modulation of autophagy affects the degradation and availability of synaptic seeding factors. This primary defect causes secondary changes to filopodial kinetics and synapse formation.

To validate the primary defect, we expressed GFP-tagged versions of the synaptic seeding factors Syd-1 and Liprin-α, and analyzed their restricted localization to synaptogenic filopodia. We use GFP-tagged versions of both proteins that we have previously shown to not affect development or function of fly photoreceptors^14^; furthermore, we validated the function of the tagged proteins by using them to rescue their respective null mutant phenotypes in photoreceptors (Supplementary Fig. 12). Using these probes, we found that autophagy-deficient terminals contain two to three times more synaptogenic filopodia with synaptic seeding factors compared with control; conversely, upregulation of autophagy leads to reduction of seeding factors in filopodia (Fig. 6i–k). In addition, the majority of Atg8a-positive autophagosomes present at filopodia tips colocalizes with with Syd-1 and Liprin-α (Supplementary Fig. 13a–c). Previous work in primary vertebrate neuronal culture as well as *Drosophila* R1–R6 photoreceptors has shown that autophagosomes formed at axon terminals traffic retrogradely to the cell body^29,49^. We therefore analyzed photoreceptor cell bodies and detected large Atg8a-positive compartments containing Syd-1 and Liprin-α (Supplementary Fig. 13d–d”). We previously implicated the upstream receptor Lar and the downstream signaling protein Trio in the kinetic regulation of synaptogenic filopodia^14^. Of these, we only detected the cytosolic protein Trio inside Atg8a-positive compartments, but not the transmembrane receptor Lar, suggesting differential availability to autophagosomal engulfment (Supplementary Fig. 13e, f’). Together, these findings indicate that autophagy controls the amount of synaptic seeding factors in filopodia, while degradation may occur during axonal transport and in cell bodies (Supplementary Fig. 13g).

### Autophagy sets a global threshold for kinetic restriction

Autophagy-dependent filopodial kinetics and synapse formation could lead to synapses with incorrect partners through at least two mechanisms. In one scenario, autophagy could be triggered only in specific filopodia, e.g., based on a molecular signal for a contact with an incorrect partner neuron in a wrong layer. Loss of autophagy would then lead to a defect in the specific removal of incorrect synapses. In support of this idea, specific presynaptic proteins have recently been shown to induce autophagy at specific places in the presynapse^50,51^. Alternatively, autophagy could set a global threshold for kinetic restriction for the entire axon terminal, such that only synaptic partners with sufficient spatial availability and molecular affinity can form synapses.

**Fig. 7:**
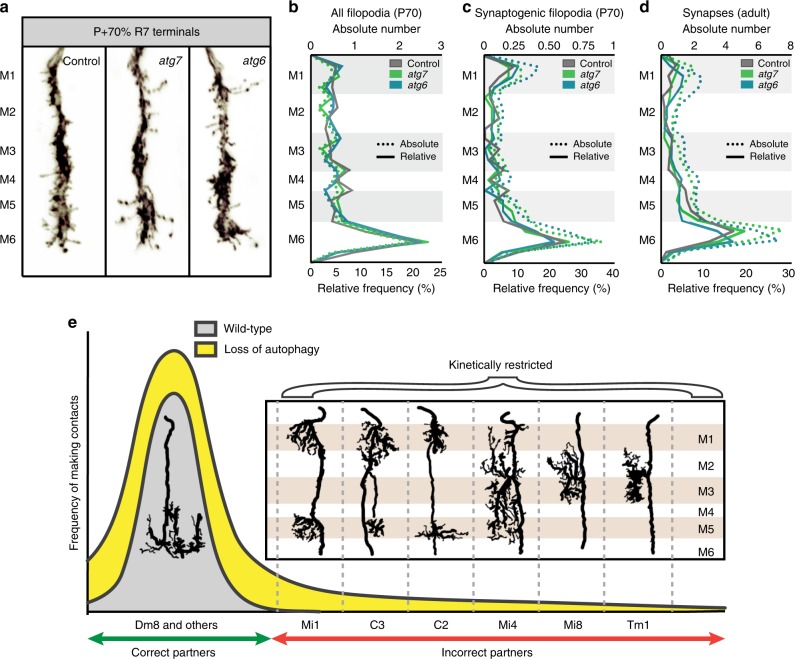
Loss of autophagy recruits incorrect synaptic partners by lowering an axon terminal-wide threshold for kinetic restriction of synapse formation. **a** Representative R7 axon terminals at P + 70% with medulla layer information. Note that the edge of medulla (M0) is defined as 0 and the end of M6 layer is defined as 100 to calculate relative positions of all filopodia and bulbous tip filopodia, and distributed to medulla layers (M1–M6) using the relative thickness of medulla layers defined by Fischbach and Dittrich^46^. Repeated five to ten times independently with similar results. **b**–**d** Relative frequency (solid lines) and absolute numbers (dotted lines) of all filopodia at P + 70% (**b**), synaptogenic filopodia at P + 70% (**c**), and synapses at 0-day-old adult (**d**). M1–M6 denote medulla layers. *n* = 40 terminals per condition. **e** Model: loss of autophagy during synaptogenesis increases the probability distribution (yellow area) compared with wild-type (gray area) of forming connections with postsynaptic partners through increased filopodial stability. Note that cells with projections at medulla layers where R7s form most of their synapses (Mi1, Mi4, C2, C3) incorrectly synapse with R7s, with higher probability than the cells with projections at medulla layers where R7s form a few, if any, synapses (Mi8, Tm1) (see Fig. 3e, f). Redrawn and adapted based on Golgi impregnations from Fischbach and Dittrich^46^. Source data are provided as a Source Data file.

To distinguish between these two models, we quantified the relative increases of all filopodia, synaptogenic filopodia, and synapses along the R7 axon terminal in medulla layers M1–M6 (Fig. 7a–d). Loss of either *atg6* or *atg7* increases the absolute numbers of synaptogenic filopodia and synapses in all medulla layers equally ~1.5-fold (dotted lines in Fig. 7b–d). As a result, the relative levels of synaptogenic filopodia and synapses between layers M1–M6 remain the same as in wild type (solid lines in Fig. 7b–d). These data indicate that autophagy is not differentially triggered in filopodia in specific medulla layers. Instead, loss of autophagy equally increases the stability of synaptogenic filopodia across the R7 terminal, resulting in the stabilization of only few filopodia in layers with low baseline filopodial activity and more pronounced increases in layers with higher baseline filopodial activity. Conversely, destabilization of filopodia along the entire R7 axon terminal in wild type effectively excludes synapse formation in layers with few filopodia, e.g., in layer M2 (Fig. 7a–d). We conclude that autophagy levels set a threshold for kinetic restriction across the R7 axon terminal.

The threshold for kinetic restriction effectively excludes synapse formation with at least six potential postsynaptic partners that are not otherwise prevented from forming synapses with R7 (Fig. 7e). We note that the localization of the presumptive dendritic trees of these six neuron types correlates well with the probabilities to be incorrectly recruited as postsynaptic partners (Figs. 3f and 7e). We speculate that specificity arises through a combination of context-dependent molecular interactions, positional effects, and kinetic restriction rather than any single factor.

## Discussion

Brain wiring requires synaptic partner choices that are both specific and robust in time and space^52^. To what extent spatiotemporal vicinity of potential partner neurons facilitates or determines partner choice remains unclear. Our findings suggest that spatiotemporal vicinity is restricted by filopodial kinetics, and that axon terminal autophagy functions as a modulator of these dynamics. Hence, kinetic restriction of synaptogenic filopodia is a means to effectively exclude synapse formation with incorrect partners (Fig. 7e). Conversely, increased stabilization of synaptogenic filopodia is sufficient to recruit as synaptic partners a surprisingly varied population of interneurons that have the principle capacity to form synapses with R7 axon terminals. At least Mi1, Mi4, C3, C2, Mi8, and Tm1 neurons in medulla columns are not prevented by “molecular mismatch” from forming synaptic contacts with R7 in vivo.

Our findings suggest that kinetic restriction sharpens synaptic specificity based on promiscuous synapse formation. Numerous studies have shown that neurons in ectopic locations readily form synapses with incorrect partners, including themselves^6,7,53^. On the other hand, Mi1, Mi4, C3, C2, Mi8, and Tm1 are all likely to express different cell surface proteins that may bias the likelihood of synaptic contacts^10,16,54^. Our data suggest that R7 terminals can form synapses with these incorrect partners simply by slowing down and stabilizing filopodial interactions. We conclude that axonal and dendritic interaction dynamics may greatly facilitate, or restrict, what partner neurons get “to see each other” and initiate synapse formation. This model requires a certain level of promiscuity in the ability to form synapses, while still being consistent with the idea of biasing certain interactions over others based on molecular interactions^1^. Recent evidence highlighted the importance of positional strategies for synaptic partner choice prior to such molecular interactions^7,11,53^. Here we have shown that positional effects are dynamic and subject to stabilization kinetics, not only when and where neuronal processes can be seen in fixed preparations. We propose that an “instruction” for synapse formation may be the product of the composite action of several factors that by themselves appear “permissive” and affect when and where neuronal surfaces meet. For example, positioning and interaction kinetics that are regulated by autophagy restrict which cell surfaces get to engage in adhesive or repellent interactions. Hence, synaptic specificity can emerge from the context-dependent combination of molecular interactions with a cell biological mechanism such as autophagy, which by itself carries no synaptic specificity information. We speculate that different neuronal thresholds for kinetic restriction can critically contribute to sharpen specificity as part of the brain’s developmental growth program.

Our findings suggest a novel role for developmental autophagy in synapse formation and brain wiring. Specifically, we report that autophagy indiscriminately destabilizes R7 synaptogenic filopodia in a manner consistent with the local degradation of a limiting resource of proteins required for synapse formation. Specificity of autophagic degradation can be triggered through interactions with proteins that themselves serve as cargo or restrict the time and place where potentially less specific engulfment occurs^19,50,51^. The bulbous tips of synaptogenic filopodia are a small space that may be easily destabilized through autophagic engulfment of proteins and other cargo, even if that engulfment were to occur in a non-selective manner. We therefore speculate that a putative cargo-specificity of autophagy may not be a prerequisite for the developmental function of autophagy described here.

Autophagy occurs at axon terminals of adult neurons and is required for neuronal maintenance in many neurons, including *Drosophila* photoreceptors^19,31,55,56^. We currently do not know to what extent developmental autophagy and autophagy during neuronal maintenance share the same initiation signals or cargo (un-)specificity. The exclusivity with which autophagosomes form in the tips of synaptogenic filopodia of developing R7 axon terminals suggest a locally restricted trigger that may well be distinct from those found in axon terminals of mature neurons. Given similar roles of autophagy in neuronal maintenance, we think it is likely to be that our observation of a specific role for developmental autophagy in the regulation of filopodial kinetics in *Drosophila* hints at similar roles in other animals and may partially explain the supernumerary dendritic spines observed in mice previously^27^.

We have previously shown that spatiotemporally regulated membrane receptor degradation is required for synapse-specific wiring in the *Drosophila* visual system^57^. Degradation and turnover of receptors and synaptic building material restrict synapse formation and contribute to specificity in a context-dependent manner. Developmentally regulated protein synthesis, trafficking, and degradation are likely to differ for different proteins and neurons at different points in time and space, where they form part of composite instructions during the growth program that give rise to specificity.

Based on this combinatorial model for specificity, we speculate that many mutations and single-nucleotide polymorphisms in the genome can result in small cell biological changes that differentially affect neurons during brain wiring. The changes effected through such modulatory, “permissive” mechanisms may not be predictable at the level of circuit wiring and behavior, yet they can cause meaningful changes to behavior that are both selectable and heritable, and thus a means of evolutionary programming of neural circuits.

## Methods

### *Drosophila* husbandry and strains

Flies were reared at 25 °C on standard cornmeal/yeast diet unless stated otherwise. For developmental analyses, white pre-pupae (P + 0%) were collected and incubated at 25 °C to pupal stages stated on figures. The following *Drosophila* strains were either obtained from Bloomington *Drosophila* Stock Center (BDSC) or other groups: *atg6*^1^ and UAS-Atg6.ORF.3xHA (E.H. Baehrecke); *atg7*^d4^ (T.Neufeld); *atg18a*^KG03090^, UAS-Brpshort-GFP, UAS-Syd-1-GFP, and UAS-Liprinα-GFP (S. Sigrist); *Trans*-tango flies (G. Barnea); GRASP flies (BDSC); ey3.5flp, GMRflp, GMR-Gal4, FRT42D, FRT80B, FRT82B, GMR-Gal80, tub-Gal80, UAS-CD4-tdGFP, UAS-CD4-tdtomato, UAS-GFP-Atg5, UAS-GFP-Atg8a, UAS-mCherry-Atg8a, UAS-Atg5RNAi (VDRC, 104461), UAS-Atg16RNAi (VDRC, 105993), GMR22F08-LexA (C2-specific driver), GMR49B06-LexA (Mi4-specific driver), and GMR19F01-LexA (Mi1-specific driver) (BDSC).

### *Drosophila* genotypes

Figure 1a–i: Controls: ey3.5flp; FRT42D/FRT42D, Cl^w+^, ey3.5flp; GMR-Gal4/+; FRT82B/FRT82B, Cl^w+^, ey3.5flp; GMR-Gal4/+; FRT80B/FRT80B, Cl^w+^ *atg7*: ey3.5flp; FRT42D, *atg7*^d4^/FRT42D, Cl^w+^, *atg6*: ey3.5flp;GMR-Gal4/+; FRT82B, *atg6*^1^/FRT82B, Cl^w+^, *atg18a*: ey3.5flp;GMR-Gal4/+; FRT80B, *atg18a*^KG03090^/FRT80B, Cl^w+^ *atg6*, GMR>Atg6: ey3.5flp;GMR-Gal4/+; FRT82B, *atg6*^1^, UAS-Atg6.ORF.3xHA /FRT82B, Cl^w+^.

Figure 2a–h: Controls: GMRflp; FRT42D, GMR-Gal80/FRT42D; GMR-Gal4, UAS-CD4-tdtomato/UAS-Brp^short^-GFP, GMRflp; GMR-Gal4, UAS-CD4-tdtomato/UAS-Brp^short^-GFP; FRT80B/FRT80B, tub-Gal80, GMRflp; GMR-Gal4, UAS-CD4-tdtomato/UAS-Brp^short^-GFP; FRT82B/FRT82B, tub-Gal80, *atg7*: GMRflp; FRT42D, GMR-Gal80/FRT42D, *atg7*^d4^; GMR-Gal4, UAS-CD4-tdtomato/UAS-Brp^short^-GFP, *atg6*: GMRflp; GMR-Gal4, UAS-CD4-tdtomato/UAS-Brp^short^-GFP; FRT82B, *atg6*^1^/FRT82B, tub-Gal80, *atg18a*: GMRflp; GMR-Gal4, UAS-CD4-tdtomato/UAS-Brp^short^-GFP; FRT80B, *atg18a*^KG03090^/FRT80B, tub-Gal80, *atg6*, GMR > Atg6: GMRflp; GMR-Gal4, UAS-CD4-tdtomato/UAS-Brp^short^-GFP; FRT82B, *atg6*^1^, UAS-Atg6.ORF.3xHA/FRT82B, tub-Gal80.

Figure 3a–g: Control: GMRflp/UAS-myrGFP, QUAS-mtdtomato(3xHA); Rh4-Gal4/*trans*-Tango; FRT82B/FRT82B, tub-Gal80, *atg6*: GMRflp/UAS-myrGFP, QUAS-mtdtomato(3xHA); Rh4-Gal4/*trans*-Tango; FRT82B, *atg6*^1^/FRT82B, tub-Gal80, *atg18a*: GMRflp/UAS-myrGFP, QUAS-mtdtomato(3xHA); Rh4-Gal4/*trans*-Tango; FRT80B, *atg18a*^KG03090^/FRT80B, tub-Gal80.

Figure 4a–c’: Control: GMRflp; Rh4-Gal4, UAS-nSyb::splitGFP1-10, LexAop-splitGFP11::GFP/ GMR19F01-LexA (Mi1) or GMR22F08-LexA (C2) or GMR49B06-LexA (Mi4); FRT82B/FRT82B, tub-Gal80. **d-f’**, *atg6*: GMRflp; Rh4-Gal4, UAS-nSyb::splitGFP1-10, LexAop-splitGFP11::GFP/ GMR19F01-LexA (Mi1) or GMR22F08-LexA (C2) or GMR49B06-LexA (Mi4); FRT82B, *atg6*^1^/FRT82B, tub-Gal80. **g-i’**, *atg18a*: GMRflp; Rh4-Gal4, UAS-nSyb::splitGFP1-10, LexAop-splitGFP11::GFP/ GMR19F01-LexA (Mi1) or GMR22F08-LexA (C2) or GMR49B06-LexA (Mi4); FRT80B, *atg18a*^KG03090^/FRT80B, tub-Gal80.

Figure 5a: GMRflp; FRT42D, GMR-Gal80/FRT42D; GMR-Gal4, UAS-CD4-tdtomato/UAS-GFP-Atg5. **b-g**, Controls: GMRflp; FRT42D, GMR-Gal80/FRT42D; GMR-Gal4, UAS-CD4-tdGFP, GMRflp; GMR-Gal4, UAS-CD4-tdGFP; FRT82B, tub-Gal80/FRT82B, *atg7*: GMRflp; FRT42D, *atg7*^d4^/FRT42D, tub-Gal80; GMR-Gal4, UAS-CD4-tdGFP, *atg6*: GMRflp; GMR-Gal4, UAS-CD4-tdGFP; FRT82B, *atg6*^1^/FRT82B, tub-Gal80, *atg6*, GMR > Atg6: GMRflp; GMR-Gal4, UAS-CD4-tdGFP; FRT82B, *atg6*^1^, UAS-Atg6.ORF.3xHA/FRT82B, tub-Gal80.

Figure 6a, b: Controls: GMRflp; FRT42D, GMR-Gal80/FRT42D; GMR-Gal4, UAS-CD4-tdGFP, GMRflp; GMR-Gal4, UAS-CD4-tdGFP; FRT82B, tub-Gal80/FRT82B, *atg7*: GMRflp; FRT42D, *atg7*^d4^/FRT42D, GMR-Gal80; GMR-Gal4, UAS-CD4-tdGFP, *atg6*: GMRflp; GMR-Gal4, UAS-CD4-tdGFP; FRT82B, *atg6*^1^/FRT82B, tub-Gal80, *atg6*, GMR > Atg6: GMRflp; GMR-Gal4, UAS-CD4-tdGFP; FRT82B, *atg6*^1^, UAS-Atg6.ORF.3xHA/FRT82B, tub-Gal80. **c**, Control: GMRflp; FRT42D/FRT42, GMR-Gal80; GMR-Gal4, UAS-CD4-tdtomato, UAS-Brp^short^-GFP, *atg7*: GMRflp; FRT42D, *atg7*^d4^/FRT42, GMR-Gal80; GMR-Gal4, UAS-CD4-tdtomato, UAS-Brp^short^-GFP, *atg6*: GMRflp; GMR-Gal4, UAS-CD4-tdtomato, UAS-Brp^short^-GFP; FRT82B, *atg6*^1^/FRT82B, tub-Gal80, *atg6*, GMR > Atg6: GMRflp; GMR-Gal4, UAS-CD4-tdtomato/UAS-Brp^short^-GFP; FRT82B, *atg6*^1^, UAS-Atg6.ORF.3xHA/FRT82B, tub-Gal80. **i-k**, Controls: GMRflp; FRT42D, UAS-Liprin-α-GFP or UAS-Syd-1-GFP/FRT42D, GMR-Gal80; GMR-Gal4, UAS-CD4-tdtomato, GMRflp; GMR-Gal4, UAS-CD4-tdtomato, UAS-Liprin-α-GFP or UAS-Syd-1-GFP; FRT82B/FRT82B, tub-Gal80, *atg7*: GMRflp; FRT42D, *atg7*^d4^, UAS-Liprin-α-GFP or UAS-Syd-1-GFP/FRT42D, tub-Gal80; GMR-Gal4, UAS-CD4-tdtomato, *atg6*: GMRflp; GMR-Gal4, UAS-CD4-tdtomato, UAS-Liprin-α-GFP or UAS-Syd-1-GFP; FRT82B, *atg6*^1^/FRT82B, tub-Gal80; *atg6*, GMR > Atg6: GMRflp; GMR-Gal4, UAS-CD4-tdtomato/ UAS-Liprin-α-GFP or UAS-Syd-1-GFP; FRT82B, *atg6*^1^, UAS-Atg6.ORF.3xHA/FRT82B, tub-Gal80.

Figure 7a–c: Controls: GMRflp; FRT42D, GMR-Gal80/FRT42D; GMR-Gal4, UAS-CD4-tdGFP, GMRflp; GMR-Gal4, UAS-CD4-tdGFP; FRT82B, tub-Gal80/FRT82B, *atg7*: GMRflp; FRT42D, *atg7*^d4^/FRT42D, GMR-Gal80; GMR-Gal4, UAS-CD4-tdGFP, *atg6*: GMRflp; GMR-Gal4, UAS-CD4-tdGFP; FRT82B, *atg6*^1^/FRT82B, tub-Gal80. **d**, Control: GMRflp; FRT42D/FRT42, GMR-Gal80; GMR-Gal4, UAS-CD4-tdtomato, UAS-Brp^short^-GFP, *atg7*: GMRflp; FRT42D, *atg7*^d4^/FRT42, GMR-Gal80; GMR-Gal4, UAS-CD4-tdtomato, UAS-Brp^short^-GFP, *atg6*: GMRflp; GMR-Gal4, UAS-CD4-tdtomato, UAS-Brp^short^-GFP; FRT82B, *atg6*^1^/FRT82B, tub-Gal80.

### Immunohistochemistry and fixed imaging

Pupal and adult eye–brain complexes were dissected in cold Schneider’s *Drosophila* medium and fixed in 4% paraformaldehyde in phosphate-buffered saline for 40 min. Tissues were washed in Phosphate-buffered saline + 0.4% Triton-X (PBST) and mounted in Vectashield (Vector Laboratories, CA). Images were obtained with a Leica TCS SP8-X white laser confocal microscope with a ×63 glycerol objective (c = 1.3). The primary antibodies used in this study with given dilutions were as follows: mouse monoclonal anti-Chaoptin (1:200; Developmental Studies Hybridoma Bank); rat monoclonal anti-nCadherin (1:100; Developmental Studies Hybridoma Bank); rabbit monoclonal anti-Atg8 (1:100; Abcam); mouse monoclonal anti-Trio (1:50; Developmental Studies Hybridoma Bank); mouse monoclonal anti-LAR (1:50; Developmental Studies Hybridoma Bank); goat polyclonal anti-GFP (1:1000; Abcam); rat monoclonal anti-GFP (1:500; BioLegend); rabbit polyclonal anti-CD4 (1:600; Atlas Antibodies); rabbit polyclonal anti-DsRed (1:500; ClonTech); rabbit anti-Syd-1 (1:500; gift from Sigrist Lab). The secondary antibodies Cy3, Cy5 (Jackson ImmunoResearch Laboratories) and Alexa488 (Invitrogen) were used in 1:500 dilution.

### Brain culture and live imaging

For all ex vivo live imaging experiments an imaging window cut open removing posterior head cuticle partially. The resultant eye–brain complexes were mounted in 0.4% dialyzed low-melting agarose, covered with a round cover slip stationed on spacers in a culture dish and let it solidify for 15 mins. Modified culture medium was added fully immersing eye–brain complexes and cover slip was sealed with glue on the edges^13^. After 45 mins of incubation at room temperature live imaging was performed using a Leica SP8 MP microscope with a 40X IRAPO water objective (numerical aperture = 1.1) with a Chameleon Ti:Sapphire laser and Optical Parametric Oscillator (Coherent). For single-channel CD4-tdGFP imaging the excitation laser was set to 900 nm and for two-color GFP/tomato imaging lasers were set to 890 nm (pump) and 1090 nm (OPO).

### *Trans*-tango and activity-dependent GRASP

For both *trans*-tango and GRASP experiments, mosaic control and autophagy-deficient R7 photoreceptors were generated by mosaic analysis with a repressible cell marker (MARCM) using the combination of GMRflp and R7-specific driver Rh4-Gal4 (see “*Drosophila* genotypes” section for detailed genotypes). *Trans*-tango flies were raised at 25 °C and transferred to 18 °C on the day of eclosion^41^. After 1 week of incubation at 18 °C, brains were dissected and stained using a standard antibody staining protocol to label postsynaptic neurons of R7 photoreceptors. The number of postsynaptic neurons was counted manually from their cell bodies using cell counter plugin in Fiji including all cell bodies with weak or strong labeling to reveal all potential connections. For activity-dependent GRASP experiments, flies were transferred to UV-transparent Plexiglas vials on the day of eclosion and kept in a custom-made light box with UV light (25 °C, 20-4 light–dark cycle) for 3 days to activate UV-sensitive R7 photoreceptors. Brains were dissected and stained with a polyclonal anti-GFP antibody to label R7 photoreceptors, monoclonal anti-GFP antibody to label GRASP signal, and polyclonal anti-CD4 antibody to label postsynaptic neurons^48^.

### Electroretinogram recordings

Newly hatched (0-day-old) adult flies were collected and glued on slides using nontoxic school glue. Flies were exposed to alternating 1 s “on” 2 s “off” light stimulus provided by computer-controlled white LED system (MC1500; Schott). ERGs were recorded using Clampex (Axon Instruments) and quantified using Clampfit (Axon Instruments).

### Buridan’s paradigm object orientation assay

Fly object orientation behavior was tested according to standard protocols using flies grown in low densities in a 12/12 h light–dark cycle^35,58^. The behavioral arena consisted of a round platform of 117 mm in diameter, surrounded by a water-filled moat and placed inside a uniformly illuminated white cylinder. The setup was illuminated with four circular fluorescent tubes (Osram, L 40w, 640 C circular cool white) powered by an Osram Quicktronic QT-M 1 × 26–42. The four fluorescent tubes were located outside of a cylindrical diffuser (DeBanier, Belgium, 2090051, Kalk transparent, 180 g, white) positioned 147.5 mm from the arena center. The temperature on the platform during the experiment was 25 °C and 30 mm-wide stripes of black cardboard were placed on the inside of the diffuser. The retinal size of the stripes depended on the position of the fly on the platform and ranged from 8.4° to 19.6° in width (11.7° in the center of the platform). Fly tracks were analyzed using CeTrAn^35^ and custom-written python code^58^. We evaluated several behavioral parameters, including center deviation and absolute distance walked, and focused on absolute stripe deviation as a parameter that gives an estimate of how precise the animals follow an object-orientated path. It is calculated as an average of all points of the fly path away from an imaginary line through the two black vertical bars. For the absolute stripe deviation, it is irrelevant whether the fly deviates to the right or left. The data was statistically analyzed using analysis of variance (ANOVA) and Tukey’s honestly significant difference (HSD) as a post-hoc test using R.

### Motion vision assay

Four-day-old female flies were immobilized on ice and glued to steel pins (7 mm × 100 μm, ENTO SPHINX s.r.o., Czech Republic) at a 60° angle from horizontal using UV-cured glue (Bondic). After a recovery time of at least 30 min, tethered flies were placed between two vertically aligned magnets. The magnetic field kept flies centered within the arena but allowed them to freely rotate 360° around their yaw-axis. For 5 min, each fly was presented with a rotating 360° panoramic pattern of green vertical bars displayed through an LED matrix (Adafruit), spanning 360° azimuthal and 45° vertical of the visual field (square wave pattern, one period = 45° azimuthal, angular velocity = 50 deg/s). An Arduino controller triggered each recording and also controlled the display of the rotating vertical bars on the LED matrix (Adafruit) surrounding the fly. Each 5 min trial consisted of consecutive iterations of the bars rotating clockwise for 5 s, stopping for 5 s and rotating counter-clockwise for 5 s. Flies were filmed from below with 60 Hz under infrared illumination (880 nm) and each fly’s body axis orientation was tracked offline using Fiji (source). For each fly the median rotational velocities during each 5 s period (CW, CCW, Stop) were calculated using circular statistics in Matlab.

### Synapse number analysis

All imaging data were analyzed and presented with Imaris (Bitplane). For synapse number analysis, CD4-tomato channel was used to generate Surfaces for individual axon terminals and Brp-positive puncta inside the Surface are filtered using the masking function. Brp-positive puncta in photoreceptor terminals were automatically detected with the spot detection module (spot diameter was set to 0.3 µm) using identical parameters between experimental conditions and corresponding controls. Synapse numbers were taken and recorded directly from statistics tab of Spot function. Graph generation and statistical analyses were done using GraphPad Prism 8.2.0

### Analyses of filopodia and synapse distributions

All imaging data were analyzed and presented with Imaris (Bitplane). For synapse distribution analysis, Brp-positive puncta were detected following the same steps in “Synapse number analysis” in R7 axon terminals. Start and endpoints of axon terminals were selected manually with the measurements point module using nCad staining as a reference (start point = beginning of nCad staining at the most distal part of medulla (M0), end point = the beginning of M7, serpentine layer in the medulla). It is noteworthy that M7 layer is devoid of synapses, hence is not labeled by nCad. The length of axon terminals are measured with the measurement point module and normalized as start point = 0 and end point = 100. The actual positions of Brp-positive puncta were exported and relative positions were calculated according to the normalized length of axon terminals. The following equation is used to calculate relative positions of Brp-positive puncta: relative position = (actual position-start point)/length × 100. For all filopodia and bulbous tip filopodia distribution analysis, the same steps were followed, except that spots were manually placed on the emerging points of all visible filopodia. Graph generation and statistical analyses were done using GraphPad Prism 8.2.0

### Filopodia tracing

Filopodia tracing was performed as previously described^14^. Briefly, we previously developed an extension to the Amira Filament Editor^59^, in which an individual growth cone is visualized as an annotated skeleton tree where each branch corresponds to a filopodium. In the first time step of four-dimensional (4D) data set, the user marks the GC center, which is automatically detected in the subsequent time steps. Filopodia tips marked by the user are automatically traced from the tip to the GC center based on an intensity-weighted Dijkstra shortest path algorithm^60^. The user visually verifies the tracing and corrects it using tools provided by the Filament Editor if necessary. After tracing all filopodia in the first time step, they are automatically propagated to the next time step with particular filopodia IDs. In every subsequent steps, the user verifies the generated tracings and adds newly emerged filopodia. This process continues until all time steps have been processed. Statistical quantities are directly extracted from the Filament Editor as spreadsheets for further data analysis.

### Mathematical modeling

*Developmental model*: We adopted the data-driven stochastic model (developmental model) from ref. ^14^. In short, the model structure remained identical, while we estimated genotype-specific parameters from the live-imaging data presented in this manuscript (Fig. 5b-e, Supplementary Movie 3, and Supplementary Table 2). In brief, we modeled synapses (S), short-lived transient bulbous tips (sB) that appeared and disappeared within the 60 min-imaging interval, and stable synaptogenic bulbous tips (synB) that persisted for more than 40 min. We also modeled two types of filopodia, which are distinguished by their lifetime and were denoted short-lived (sF) and long-lived (F) filopodia.

The model’s reaction stoichiometries are determined by the following reaction scheme:

$$R_{1,sF}:\emptyset \to sF,\quad R_{2,sF}:sF \to \emptyset ,\quad R_{1,\ell F}:\emptyset \to \ell F, \quad R_{2,\ell F}:\ell F \to \emptyset$$

$$R_3:F \to sB,\quad R_4:sB \to \emptyset ,\quad R_5:sB \to synB,\quad R_6:synB \to S$$

where reactions *R*_1,*sF*_ and *R*_1,ℓ*F*_ denote the generation of short- and long-lived filopodia, whereas *R*_2,*sF*_ and *R*_2,*ℓF*_ denote their retraction. Reaction *R*_3_ denotes the formation of a (transient) bulbous tip, whereas *R*_4_ denotes its retraction. Reaction *R*_5_ denotes the stabilization of a transient bulbous tip and, finally, a stable bulb forms a synapse with reaction *R*_6_.

It is noteworthy that in R_3_ we denote by *F* any filopodium (short-lived and long-lived) and in R_4_ we have ignored the flux back into the filopodia compartment *sF* + *ℓF*, as it insignificantly affects the number of filopodia (small number of bulbous tips, small rate *r*_4_).

Similar to the published model^14^, reaction rates/propensities of the stochastic model are given by

$$\begin{array}{*{20}{c}} {r_{1,sF}\left( t \right) = f_F\left( t \right) \cdot c_{1,sF},} & {r_{2,sF}\left( {sF} \right) = sF\cdot c_{2,sF}} \end{array}$$

$$\begin{array}{*{20}{c}} {r_{1,\ell F}\left( t \right) = f_F\left( t \right) \cdot c_{1,\ell F},} & {r_{2,\ell F}\left( {sF} \right) = \ell F\cdot c_{2,\ell F}} \end{array}$$

$$\begin{array}{*{20}{c}} {r_3\left( {t,sF,\ell F,B} \right) = c_3\left( {sF + \ell F} \right) \cdot f_1\left( {synB,B_{50}} \right) \cdot f_{FB}\left( {t,t_{\frac{1}{2}}} \right),} & {r_4\left( {sB} \right) = c_4\cdot sB} \end{array}$$

$$\begin{array}{*{20}{c}} {r_5\left( {sB} \right) = c_5 \cdot sB,} & {r_6\left( {synB} \right) = c_6 \cdot synB,} \end{array}$$

where *c*_1_…*c*_6_ are reaction constants (estimated as outlined below). The feedback function $f_1\left( {synB,B_{50}} \right) = \left( {synB + B_{50}} \right)/B_{50}$ models bulbous auto-inhibition due to limited resources and synaptic seeding factor competition as introduced before^14^. The functions *f*_*F*_(*t*) and $f_{FB}\left( {t,t_{\frac{1}{2}}} \right)$ model slow-scale dynamics of filopodia- and bulbous dynamics, with previously determined parameters^14^:

*f*_*FB*_(*t*) is a tanh function with

$f_{FB}( {t,t_{1/2}} ) = \frac{1}{2}\left( {1 + \tanh \left[ {\frac{3}{{t_{1/2}}}\left( {t - t_{1/2}} \right)} \right]} \right)$, which models a time-dependent increase in the propensity to form bulbous tips with *t*_1/2_ = 1000 (min). The time-dependent function $f_F\left( t \right) = {\mathrm{max}}\left( {0,\mathop {\sum}\nolimits_{i = 0}^5 {p_i \cdot t^i} } \right)$ is a fifth-order polynome with coefficients *p*_5_ = −2.97 × 10^−14^, *p*_4_ = 3.31 × 10^−13^, *p*_3_ = −1.29 × 10^−9^, *p*_2_ = 2.06 × 10^−6^, *p*_1_ = −1.45 × 10^−3^, and *p*_0_ = 1, which downregulates the generation of new filopodia at a slow time scale. It is noteworthy that *t* denotes the time in (min) after P40 (e.g., *t*_P40_ = 0 and *t*_P60_ = 60 × 20).

*Parameter estimation*: Using the methods explained below, we derived the parameters depicted in Supplementary Table S2. We first estimated *c*_2,*sF*_, *c*_2,ℓ*F*_ from the filopodial lifetime data, whereby *c*_2,*sF*_ was approximated as the inverse of the lifetimes of all filopodia that lived <8 min and *c*_2,*ℓF*_ from all filopodia living at least 8 min. We realized that the number of filopodia per time instance was Poisson distributed (Supplementary Fig. 9, solid black lines), i.e., *sF*~${\mathcal{P}}$(*λ*_*sF*_) and ℓ*F*~${\mathcal{P}}$(*λ*_ℓ*F*_), where *λ* denotes the average number of filopodia per time instance. Given the first-order retraction of filopodia (≈exponential lifetime), the Poisson distribution can be explained by a zero-order input with rate *c*_1,*sF*_ and *c*_1,ℓ*F*_, and *λ*_*sF*_ = *r*_1,*sF*_/*c*_2,*sF*_ and *λ*_ℓ*F*_ = *r*_1,ℓ*F*_/*c*_2,ℓ*F*_, respectively. Using the mean number of *sF*, ℓ*F* at P60 we then estimated $c_{1,sF} = \lambda _{sF}\left( {P60} \right) \cdot c_{2,sF}/f_F\left( {P60} \right)$ and $c_{1,\ell F} = \lambda _{sF}\left( {P60} \right) \cdot c_{2,\ell F}/f_F\left( {P60} \right)$.

Next, we investigated the lifetimes of bulbous tip filopodia (Supplementary Fig. 11b–e). We realized that akin to the wild type, the *atg6* and *atg7* exhibited almost no transient bulbous tips. We therefore set *c*_4_ = 1/120 (min^−1^) according to the published model^14^. Furthermore, we determined *c*_6_ from the steepest slope in Fig. 6c (control data) divided by the average number of Bulbs ($5 \approx \smallint_t^{t + {\mathrm{\Delta }}t} r_6\left( s \right)\,ds = \smallint_t^{t + {\mathrm{\Delta }}t} synB\left( s \right) \cdot c_6\,ds \Rightarrow c_6 \approx \frac{5}{{1.1 \cdot 10 \cdot 60}} = 1/133$ min^−1^). We then estimated the three parameters *c*_5_, *B*_50_, and *r*_3_(*t*) for *t* = P60. To do so, we used the number distribution of short-lived and synaptogenic bulbous tips (Fig. 5f, g), and set up the generator matrix

$${G\left( {\left[ {i,j} \right],\left[ {i - 1,j} \right]} \right) = i \cdot c_4,} \qquad\;\, {G\left( {\left[ {i,j} \right],\left[ {i,j - 1} \right]} \right) = j \cdot c_6}\qquad\qquad\\ {G\left( {\left[ {i,j} \right],\left[ {i + 1,j} \right]} \right) = r_3\left( t \right) \cdot f_1\left( {j,B_{50}} \right),} \qquad {G\left( {\left[ {i,j} \right],\left[ {i,j + 1} \right]} \right) = j \cdot c_5}\qquad\qquad\;$$

with diagonal elements such that the row sum equals 0. In the notation above, the tupel [*i*, *j*] denotes the state where *i* short-lived bulbous tips *sB* and *j* synaptogenic bulbous tips *synB* are present. The generator above has a reflecting boundary at sufficiently large *N* (maximum number of bulbous tips). Above, *r*_3_(*t*) is auto-inhibited by the number of stable bulbous tips through function *f*_1_. The stationary distribution of this model is derived by solving the eigenvalue problem

$$G^T \cdot v = v \cdot \lambda$$

and finding the eigenvector corresponding to eigenvalue *λ*_0_ = 0. From this stationary distribution, we compute the marginal densities of *sB* and *synB* (e.g., summing over all states where *i* = 0, 1,… for sB) and fit them to the experimentally derived frequencies by minimizing the Kullback–Leibler divergence between the experimental and model-predicted distributions. Lastly, parameter *c*_3_ is derived by calculating

1 $$c_3 = \frac{{r_3\left( t \right)}}{{\left( {sF\left( t \right) + \ell F\left( t \right)} \right) \cdot f_{FB}\left( {t,t_{1/2}} \right)}}$$

where *sF*(*t*) = *sF*(*t*_*p*60_), ℓ*F*(*t*) = ℓ*F*(*t*_*p*60_), and *f*_*FB*_(*t*) = *f*_*FB*_(*t*_*P*60_, *t*_1/2_).

*Mechanistic model*: This model explains autophagy mutant phenotypes as a consequence of increased seeding factor abundance. We adopted the mechanistic model from ref. ^14^. This model essentially assumes a dynamic pool of a limited resource of bulbous tip-stabilizing factors (Fig. 6g and Supplementary Fig. 11a). The model consists of four types of reactions: new filopodia emerge (reaction *G*_1_), accumulate resources (reaction *G*_2_), retract (reaction *G*_3_), or release resources (reaction *G*_4_).

$${G_1: \emptyset \to F,} \qquad\;\; {G_2:\quad FS_{i - 1} + S \to FS_i,}$$

$${G_3:\,\,FS_i \to S_i,} \qquad {G_4:\;\;FS_i \to FS_{i - 1} + S,}\quad$$

where *F* denotes an “empty” filopodium, *S* denotes the seeding factor, and *FS*_i_ denotes a filopodium with *i* seeding factor proteins in it. The reaction rates (propensities) were modeled as

$${g_1 = const,} \quad {g_2(i - 1) = FS_{i - 1} \cdot S \cdot c_{in},}$$

$${g_3\left( i \right) = FS_i \cdot \frac{1}{i},}\quad {g_4\left( i \right) = FS_i \cdot c_{out},}\quad\qquad\;\;\;$$

where we set *g*_1_ equal to the average rate of transient bulbous tip emergence in the control experiments at P60, i.e., *g*_1_ = *r*_3_(*t*_*P*60_, *WT*). Reaction rate *g*_3_ implements a competitive advantage: the lifetime of bulbous filopodia is increased proportionally to the number of seeding factors it accumulated. The parameters *c*_in_ and *c*_out_ were set to values 0.07 and 1.5 (time^−1^), and as initial condition we set $S\left( {t_0} \right) = \left\| {n \cdot \overline B \left( {t_{P60}} \right)} \right\|$, where *n* is the number of states (we used *n* = 120), $\overline B \left( {t_{P60}} \right)$ denotes the genotype-specific average number of bulbous tips at P60 and $|| \cdot ||$ denotes the next integer function.

Importantly, in the model, the wild-type and the *atg6*- and *atg7*-knockout mutants only differ in the total number of seeding factors available.

We stochastically ran the model 100,000 time steps to reach a steady state and discarded the first half as a burn-in period (pre-steady state). Subsequently, we analyzed the number of bulbous tips and their lifetimes from the remaining time steps as shown in Supplementary Fig. 11b–i. Thereby, we assumed that filopodia would be recognized as bulbous tips only if they contained at least *n*/4 seeding factors.

In summary, these computational experiments highlight that the phenotype of the *atg6*- and *atg7*-knockout mutants can be solely explained by an increased abundance of seeding factors ( = compromised ability to degrade seeding factors).

In the case of autophagy upregulation *(atg6*, GMR > Atg6), we observed a different phenotype: from the data-driven model, we could see that bulbous tips were destabilized (parameter r4 in Supplementary Table S2) and also that the feedback was lost (parameter E[f1] close to 1 in Supplementary Table S2). We tested different parameter- and model alterations to reproduce both the number and lifetime distribution of bulbous tips. Finally, we found that if seeding factors no longer stabilized bulbous tips (loss in the competitive advantage), both the lifetime and the number distribution of bulbous tips can be accurately reproduced. Thus, we set reaction rate *g*_3_ to *g*_3_ = *FS*·*const*, for autophagy upregulation, where *const* = *c*_4_ (time^−1^; Supplementary Table 3).

### Statistical analysis

Individual data in the same group were first checked for normal distribution using D’Agostino and Pearson normality test. If all distributed normal, one-way ANOVA and Tukey’s HSD as post-hoc tests were used. If at least one data shows non-normal distribution, then non-parametric Kruskal–Wallis and Dunn’s as post-hoc tests were used. All significance values are denoted on the graphs and in their respective legends. Data were analyzed using GraphPad Prism 8.2.0 (GraphPad Software, San Diego, CA).

### Lead contact and materials availability

All reagents used in this study are available for distribution. Requests for resources and reagents should be directed to Robin Hiesinger (robin.hiesinger@fu-berlin.de).

### Reporting summary

Further information on research design is available in the Nature Research Reporting Summary linked to this article.

## Supplementary information

Supplementary Information

Peer Review

Reporting Summary

Description of Additional Supplementary Files

Supplementary Movie 1

Supplementary Movie 2

Supplementary Movie 3

## Data Availability

Raw (.lif format) and processed (.ims and.am format) imaging datasets are available on request. The filopodia-tracking software is an extension of the commercial software Amira, which is available from Thermo Fisher Scientific. The filopodia-tracking software is available from the corresponding author upon request in source code and binary form. Executing the binary requires a commercial license for Amira. The source data underlying Figs. 1d, e, i, 2f, g, h, 3d, g, 5g, 6h, j, k, 7b–d and Supplementary Figs. 1e, 2d, 2f, 2g, 2i, 3b, 4b, 4d, 4h, 4l, 5b, 7b, 7c, 7e, 12f, 13c are provided as a Source Data file.

## Source Data

Source Data

## Code availability

MATLAB codes for model parameter inference for model simulation have previously been published^14^ and are available through https://github.com/vkleist/Filo2.
