# Supplementary material for: Autophagy-dependent filopodial kinetics restrict synaptic partner choice during Drosophila brain wiring
Source: Nat Commun. 2020 Mar 12;11:1325. doi: 10.1038/s41467-020-14781-4 (PMC7067798; doi:10.1038/s41467-020-14781-4)
Supplement: Supplementary file 1 — Supplementary Information [file 41467_2020_14781_MOESM1_ESM.pdf]

Supplementary Information

Autophagy-dependent filopodial kinetics restrict synaptic partner choice during *Drosophila* brain wiring

Kiral et al.

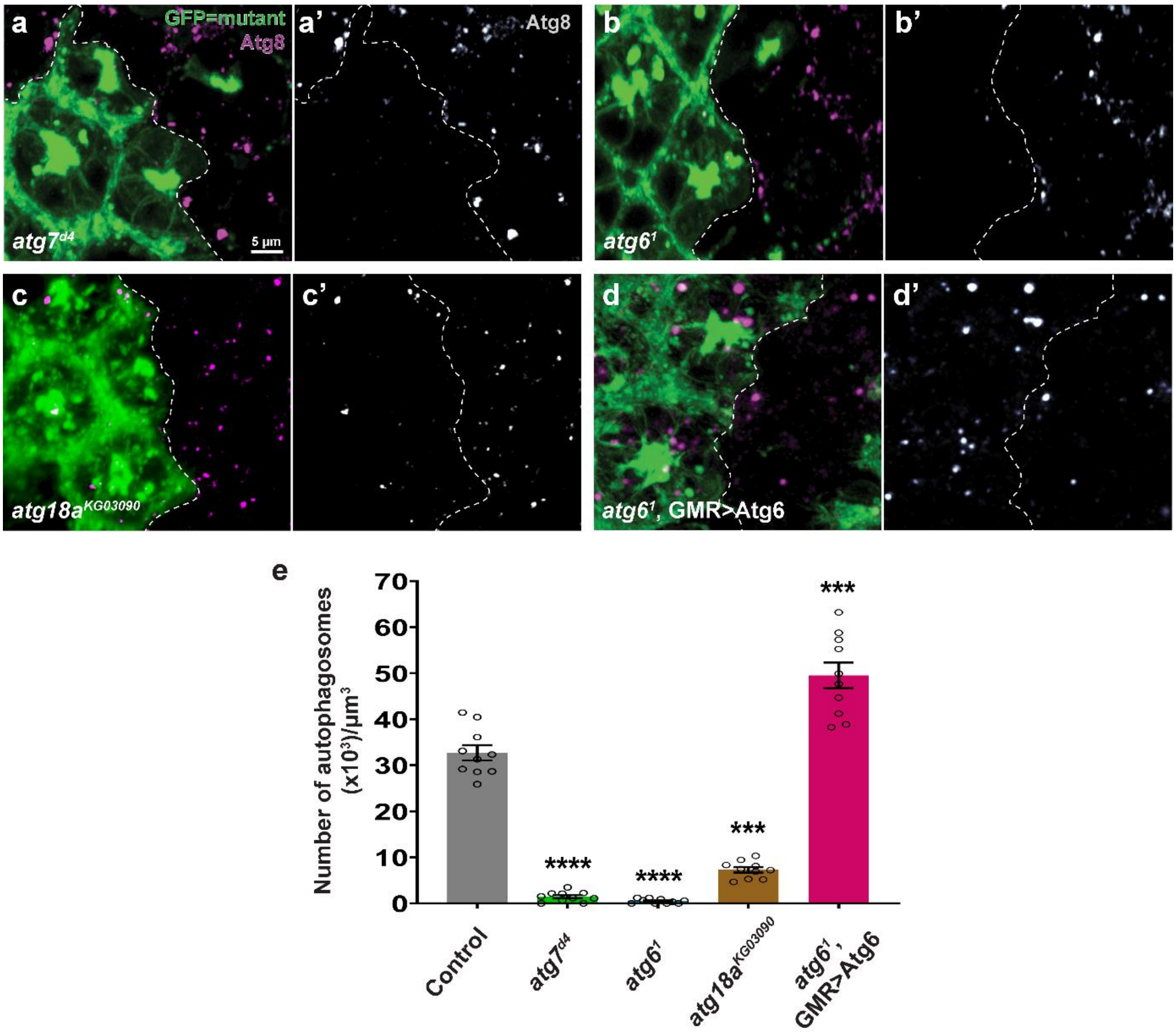

Supplementary Figure 1. Atg6, Atg7 and Atg18 are required for developmental autophagy in *Drosophila* photoreceptors. a-d', Atg8 immunolabelled autophagosomes in GFP-positive photoreceptor clones of $atg7^{d4}$ **(a-a')**, $atg6^{1}$ **(b-b')**, $atg6^{1}$, GMR>Atg6 **(c-c')** and $atg18a^{KG03090}$ **(d-d')** versus non-GFP control clones in genetic mosaics of P+50% pupal retina. Repeated 3 times independently with similar results. **e,** Number of autophagosomes in a given volume. Note almost complete abolishment of autophagosomes in $atg7^{d4}$ and $atg6^{1}$ mutant photoreceptors, milder decrease in autophagosome number in $atg18a^{KG03090}$ mutant photoreceptors and a significant increase in autophagosome number in $atg6^{1}$, GMR>Atg6 photoreceptors. n=10 retinas per condition, one region of interest is randomly selected per retina. One-way ANOVA and Tukey HSD as post hoc test; ***$p<0.001$, ****$p<0.0001$. Error bars denote mean ± SEM. Source data are provided as a Source Data file.

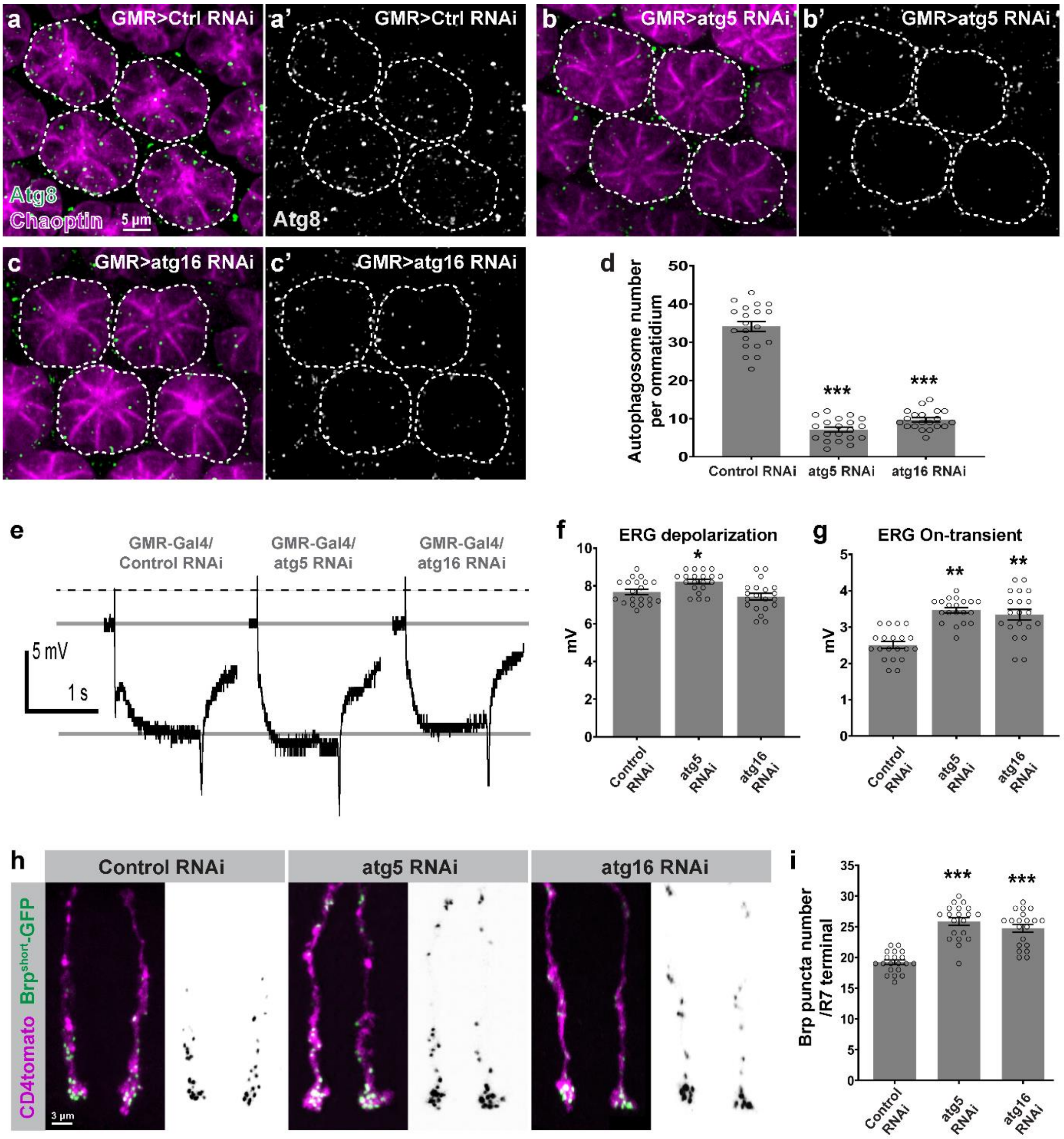

Supplementary Figure 2. Downregulation of autophagy by Atg5 and Atg16 knockdown leads to increased neurotransmission and synapse number. a-c', Atg8 labelled autophagosomes in Chaoptin labelled photoreceptor cell bodies of GMR-Gal4 driven control RNAi **(a-a')**, Atg5 RNAi **(b-b')** and Atg16 RNAi **(c-c')**. Dashed lines encircle photoreceptor cell bodies in individual ommatidium. Repeated 3 times independently with similar results. **d,** Number of autophagosomes per ommatidium. n=20 ommatidia per condition. **e,** Representative ERG traces recorded from control (GMR-Gal4/Control RNAi), Atg5 knockdown (GMR-Gal4/Atg5 RNAi) and Atg16 knockdown (GMR-Gal4/Atg16 RNAi) photoreceptors. Repeated 3 times independently with similar results. **f-g,** Quantifications of ERG depolarization (**f**) and ERG on-transient (**g**). n=20 flies per condition. **h,** Representative images of Brp^{short}-GFP labelled active zones in control, Atg5 knockdown and Atg16 knockdown R7 axon terminals. Repeated 3 times independently with similar results. **i,** Number of Brp puncta in control, Atg5 knockdown and Atg16 knockdown R7 axon terminals. n=20 terminals per condition. One-way ANOVA and Tukey HSD as post hoc test; *p<0.05, **p<0.01, ***p<0.001. Error bars denote mean ± SEM. Source data are provided as a Source Data file.

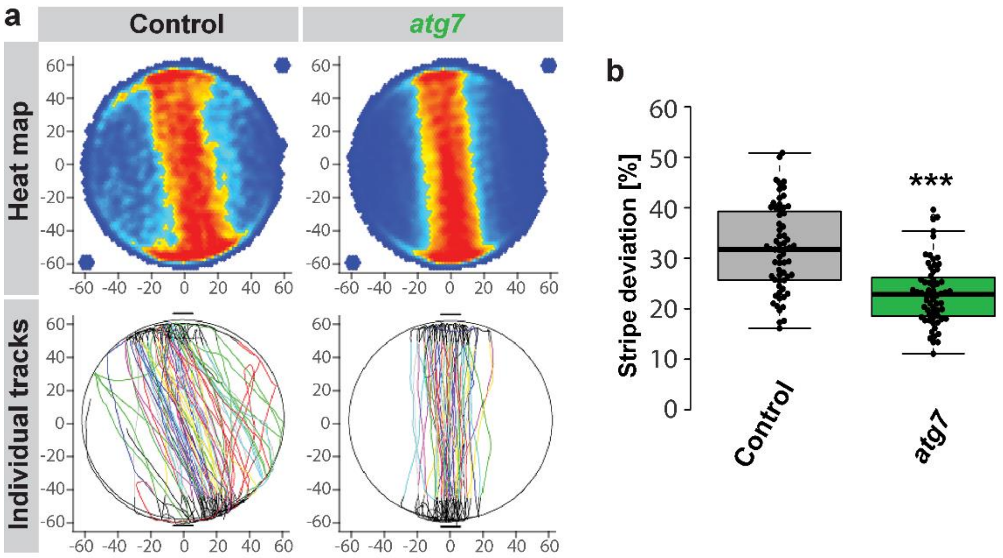

Supplementary Figure 3. Loss of *atg7* in *Drosophila* photoreceptors leads to increased visual attention behavior. a, Stripe fixation behavior of adult flies with control and *atg7* mutant photoreceptors is shown on the population level (heatmap) and as individual tracks. **b,** Quantification of stripe deviation. The bottom-most and top-most horizontal lines, the lower and upper hinges, and the middle line of the boxplots indicate the minimum and maximum values, the 25th and 75th percentiles, and the median, respectively. n=60 flies per condition, two-way ANOVA and Tukey HSD as post hoc test, ***$p<0.001$. Note that similar to flies with *atg6* mutant photoreceptors (see Fig. 1h), flies with *atg7* mutant photoreceptors show increased stripe fixation behavior and repetitive walks between stripes. Source data are provided as a Source Data file.

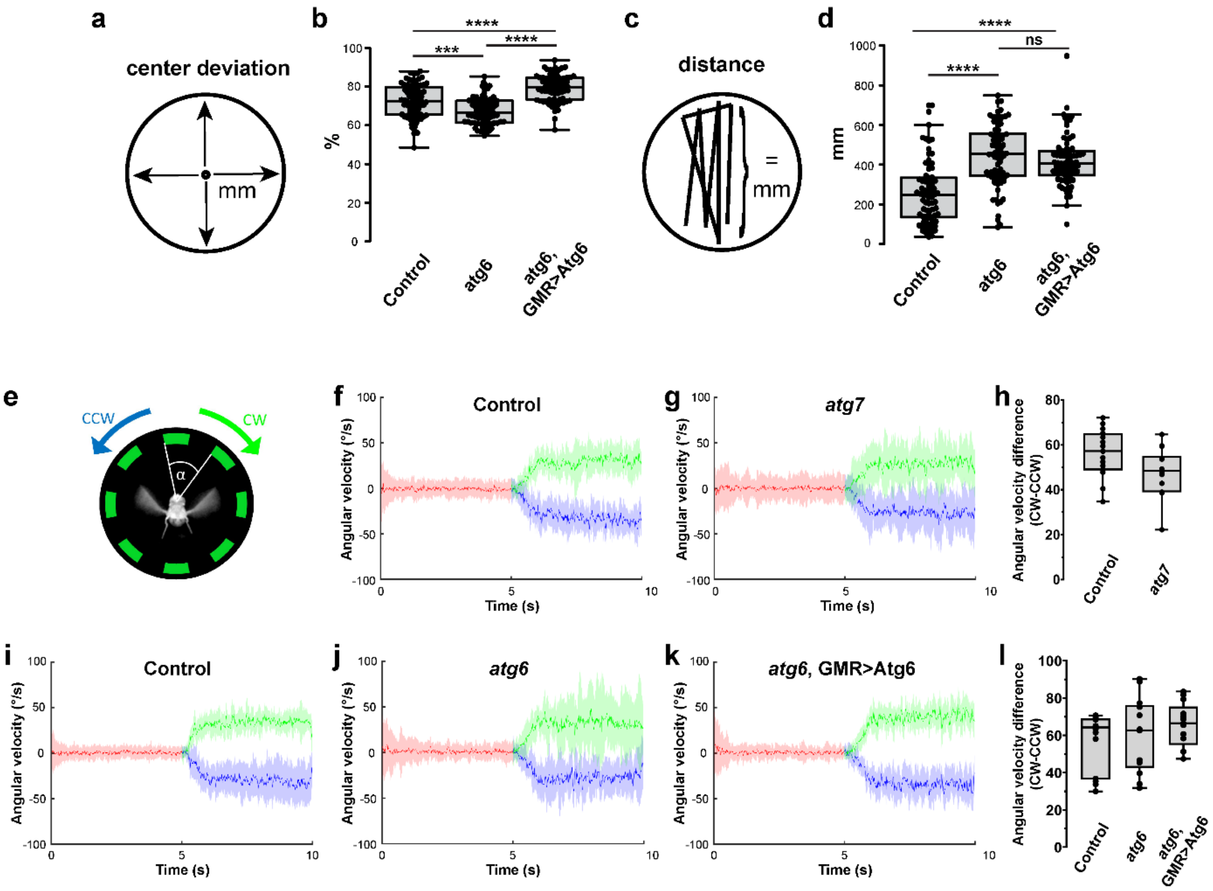

Supplementary Figure 4. Optomotor behavior is unaffected in flies deficient of autophagy in photoreceptors. a, The parameter 'center deviation' measures how much a fly deviates from the center point of the arena. **b,** Quantification of center deviation. n=60 flies per condition, two-way ANOVA and Tukey HSD as post hoc test; ***p<0.001, ****p<0.0001. **c,** The parameter 'distance' measures the total distance travelled by a fly during the imaging window. **d,** Quantification of distance. n=60 flies per condition, two-way ANOVA and Tukey HSD as post hoc test; ****p<0.0001. **e,** Schematic representation of the optomotor setup. A tethered fly is presented with moving green vertical stripes (α = 45°, rotational velocity either 0°/s, or 50°/s CCW or CW, respectively). **f-g** and **i-k**, Averaged optomotor responses of all flies of the tested groups for periods of the vertical stripes not moving (red), moving CW (green) or moving CCW (blue), respectively. Shaded areas indicate the standard deviation. **h** and **l**, Box plots depict the average difference in angular velocity (mean CW - mean CCW) for all tested flies of each individual group. n=15 (control for *atg7*), n=14 (*atg7*), n=14 (control for *atg6* and atg6, GMR>Atg6), n=13 (*atg6*), n=13 (GMR>Atg6). Wilcoxon rank sum test. No significant difference found between groups. The bottom-most and top-most horizontal lines, the lower and upper hinges, and the middle line of the boxplots indicate the minimum and maximum values, the 25th and 75th percentiles, and the median, respectively. Source data are provided as a Source Data file.

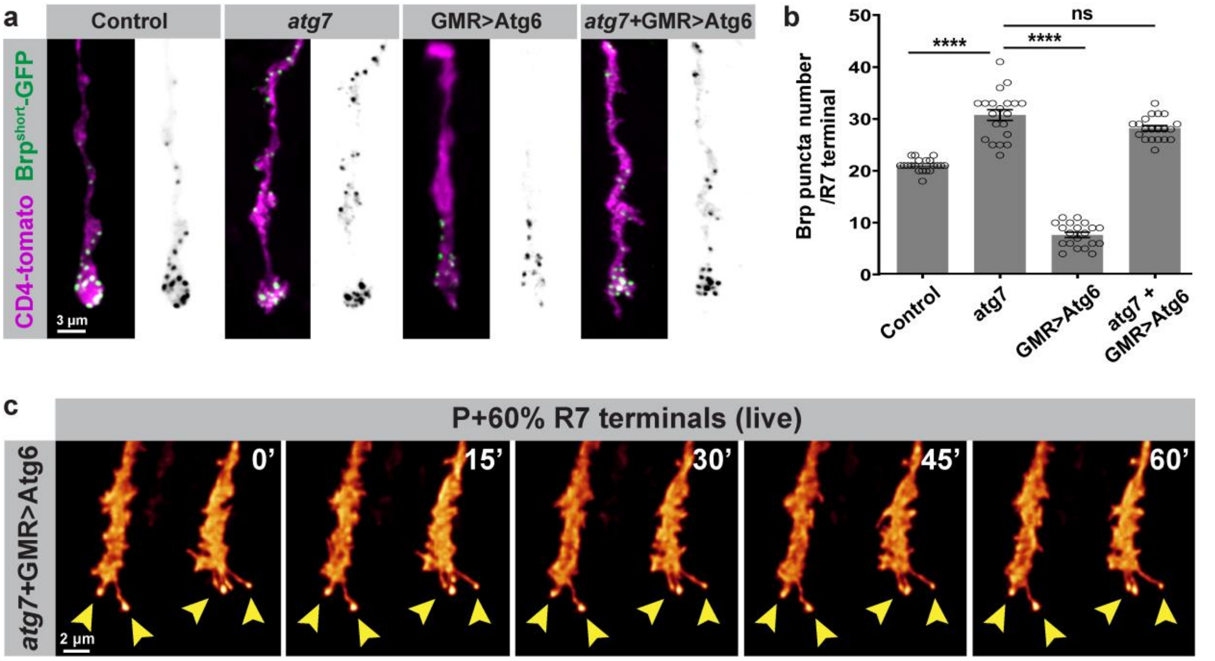

Supplementary Figure 5. Decreased filopodial stability and synapse number in Atg6 overexpression is due to upregulation of autophagy. a, Representative images of control (same as in fig.2a), *atg7*; GMR>Atg6; and *atg7*, GMR>Atg6 R7 photoreceptor axon terminals with Brp^{short-}GFP marked active zones. Repeated 5 times independently with similar results. **b,** Number of BRP puncta in control, *atg7*, GMR>Atg6, and *atg7*, GMR>Atg6 R7 photoreceptor axon terminals. n=20 terminals per condition. Kruskal-Wallis and Dunn's as post hoc test; ****$p<0.0001$. Error bars denote mean ± SEM. **c,** Live imaging of *atg7*, GMR>Atg6 R7 axon terminals at P+60%. Note that Atg6 overexpression on *atg7* mutant background still shows increased number of stable bulbous tip filopodia similar to *atg7* mutant alone (see fig. 5c). Repeated 2 times independently with similar results. Source data are provided as a Source Data file.

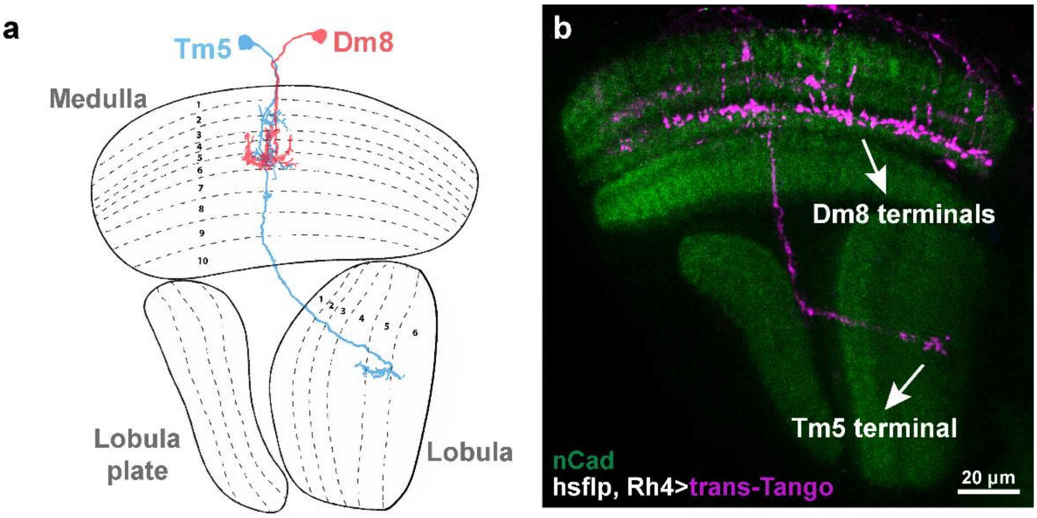

Supplementary Figure 6. Dm8 and Tm5 neurons are the main postsynaptic targets of R7 photoreceptors. a, Schematic of Dm8 and Tm5 neuronal morphology in *Drosophila* optic lobe. **b,** Sparse trans-Tango labelling protocol with R7-specific Rh4-Gal4 revealed that R7 photoreceptors mainly connect to Dm8 and Tm5 neurons in wild-type brains. Repeated 3 times independently with similar results.

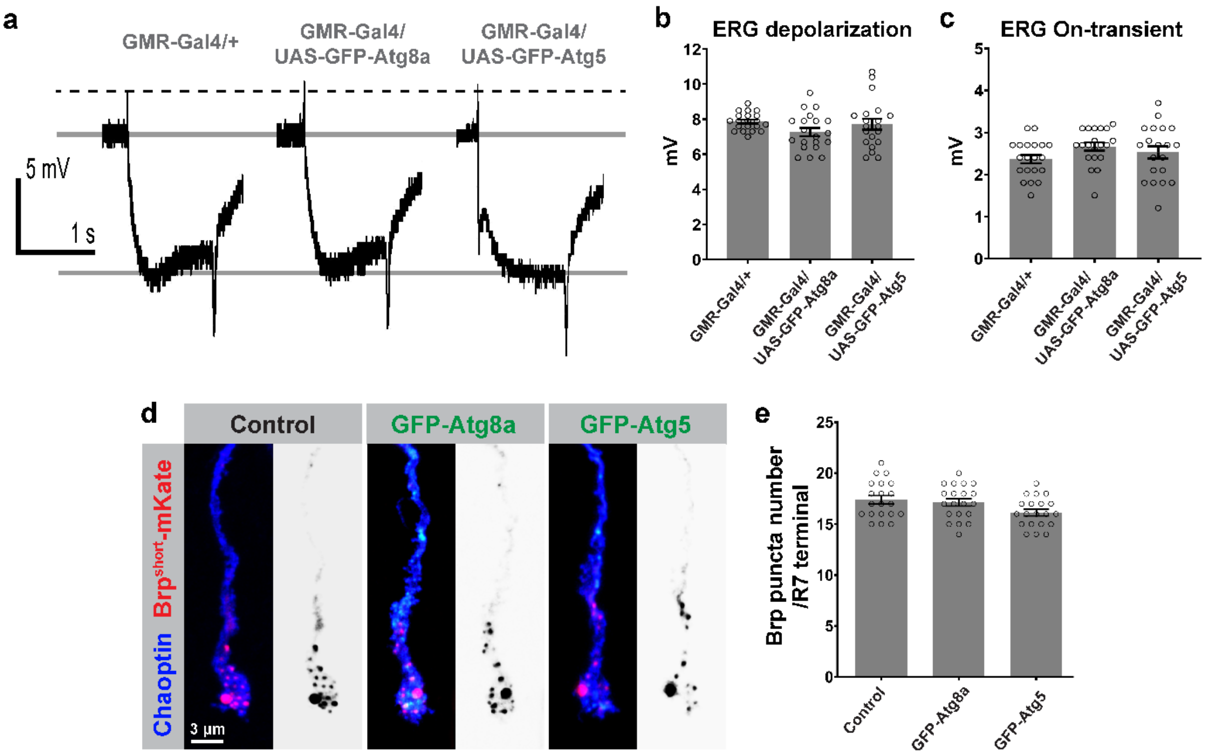

Supplementary Figure 7. Atg5 or Atg8a overexpression does not alter neurotransmission or synapse number. a, Representative ERG traces recorded from control (GMR-Gal4/+), GFP-Atg8a expressing (GMR-Gal4/GFP-Atg8a) and GFP-Atg5 expressing (GMR-Gal4/GFP-Atg5) photoreceptors. Repeated 3 times independently with similar results. **b-c,** Quantifications of ERG depolarization (**b**) and ERG on-transient (**c**). n=20 flies per condition. **d,** Representative images of Brp^{short}-mKate labelled active zones in control, GFP-Atg8a expressing, GFP-Atg5 expressing R7 axon terminals. Repeated 5 times independently with similar results. **e,** Number of Brp puncta in control, GFP-Atg8a expressing, GFP-Atg5 expressing R7 axon terminals. n=20 terminals per condition. One-way ANOVA and Tukey HSD as post hoc test. Error bars denote mean ± SEM. No significant difference found between groups. Source data are provided as a Source Data file.

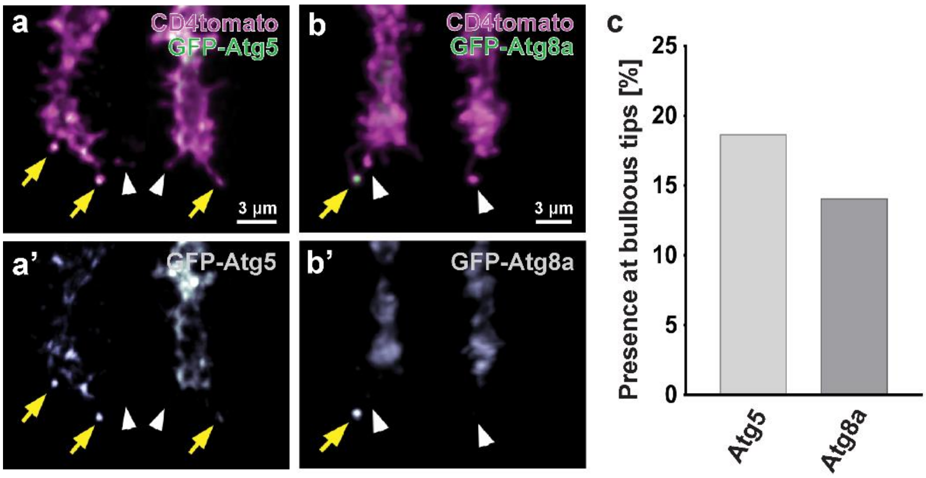

Supplementary Figure 8. The essential autophagy proteins Atg5 and Atg8a localize to synaptogenic filopodia tips. a-b', Localization of autophagy essential proteins Atg5 (**a-a'**) and Atg8a (**b-b'**) to bulbous tip filopodia (P+60%). Yellow arrows show the presence of Atg5 and Atg8a at bulbous tips, while white arrowheads show bulbous tips without Atg5 and Atg8a. Repeated 5-10 times independently with similar results. **c,** Percentage of bulbous tip filopodia with Atg5 and Atg8a signal to all bulbous tip filopodia. n=30 terminals. All bulbous tip filopodia from 30 axon terminals were pooled for quantification.

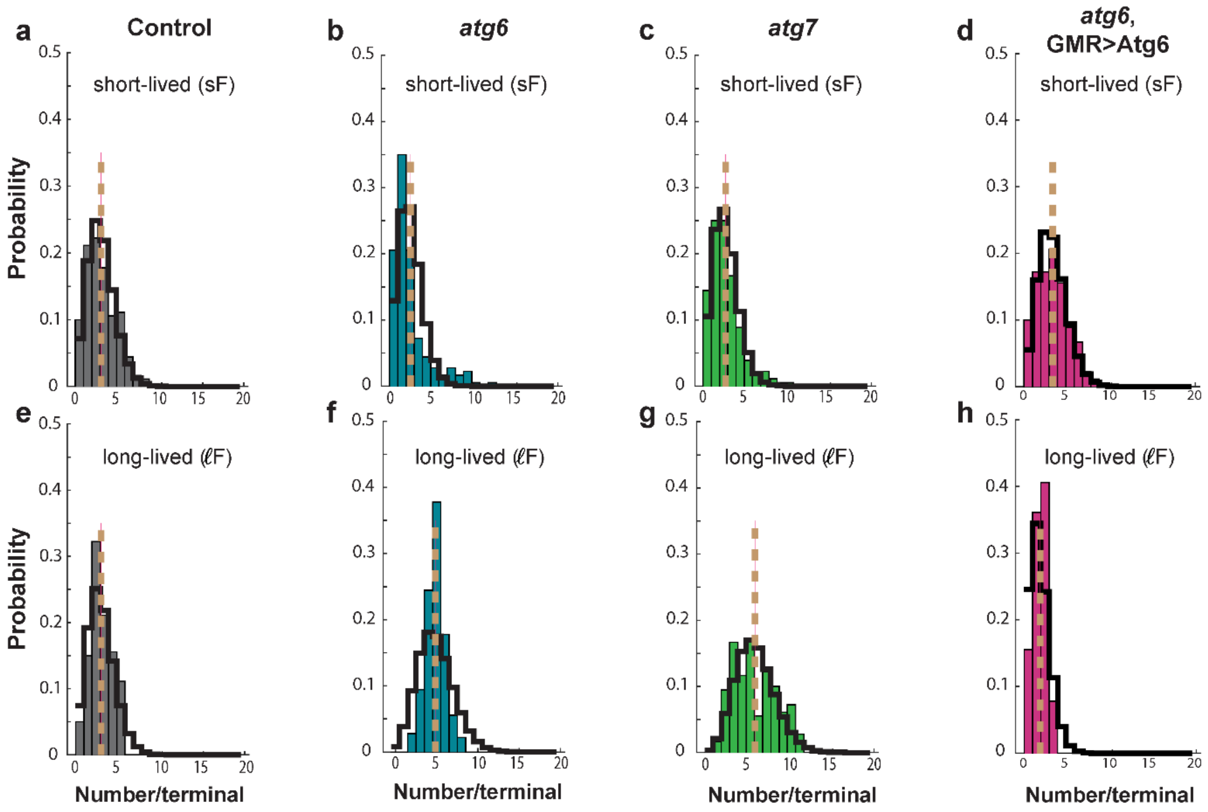

Supplementary Figure 9. Number of short-lived and long-lived filopodia at P60. Bars denote the observed numbers during live imaging and the dashed vertical line indicates the average numbers. The solid black trace depicts a Poisson distribution with expectation value equal to the average number of observed filopodia. short-lived filopodia = filopodia exist shorter than 8 mins, long-lived filopodia = filopodia exist longer than 8 mins. Values for lifetimes and numbers are shown in Supplementary Table 1.

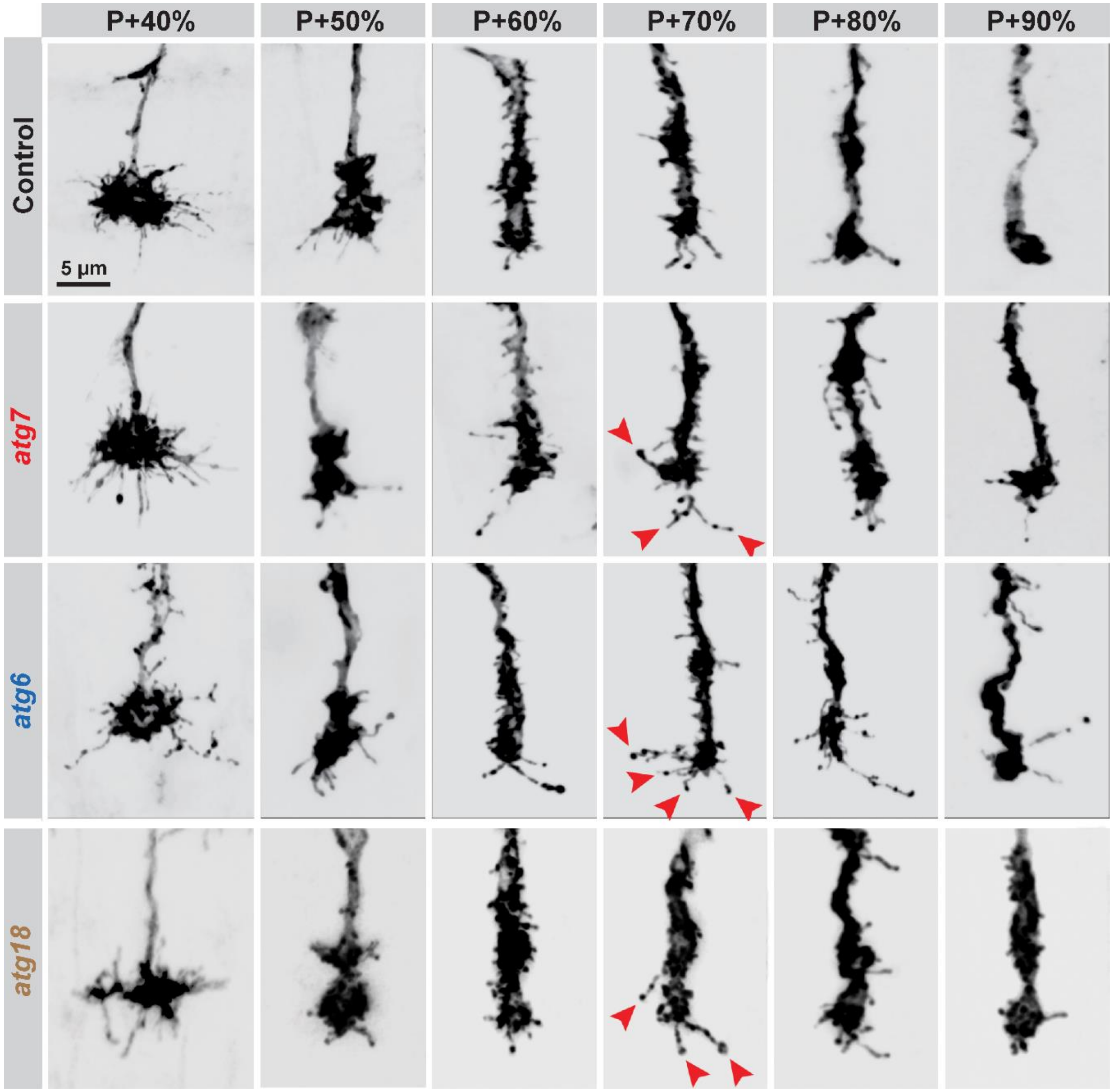

Supplementary Figure 10. Morphology of R7 photoreceptor axon terminals throughout the second half of pupation (the period of synapse formation). Representative images of control, *atg7*, *atg6* and *atg18* mutant R7 axon terminal morphologies at P+40%, P+50%, P+60%, P+70%, P+80%, and P+90% pupal development. Red arrowheads show examples of supernumerary bulbous tip filopodia at P+70%. Note that loss of autophagy leads to increased numbers of bulbous tip filopodia especially during the peak time of synaptogenesis (P+60%-P+80%). Repeated 5-10 times independently with similar results.

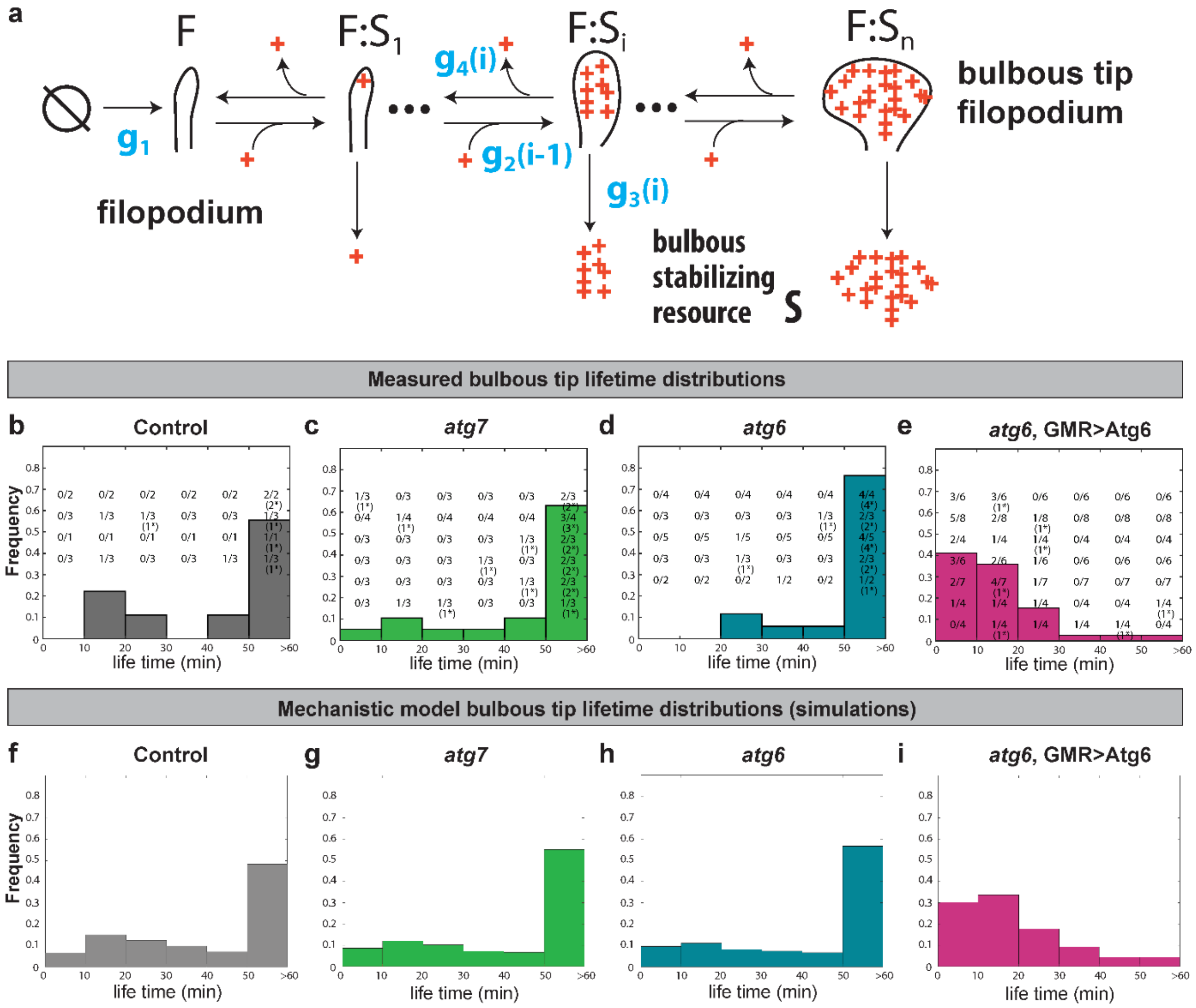

Supplementary Figure 11. The Mechanistic Model: Lifetimes of synaptogenic bulbous tip filopodia as a function of a limiting resource of synaptic seeding factors. a, Graphical depiction of the mechanistic model. **b-e,** Measured data: Histograms depicting the observed frequency of the respective bulbous tip life times during live imaging at P60. The numbers on histograms indicate the number of observations in the respective life time category per growth cone. Numbers in brackets with a star, e.g. (1*), indicate that the bulbous tip either already existed in the first imaging frame, or persisted until the last image. Thus, these life times might actually be longer than indicated here. **f-i,** Model output: Histograms depicting the frequency of the respective bulbous tip lifetimes according to simulations using the mechanistic model. Note that the mechanistic model successfully recapitulates the observed lifetimes of bulbous tip filopodia.

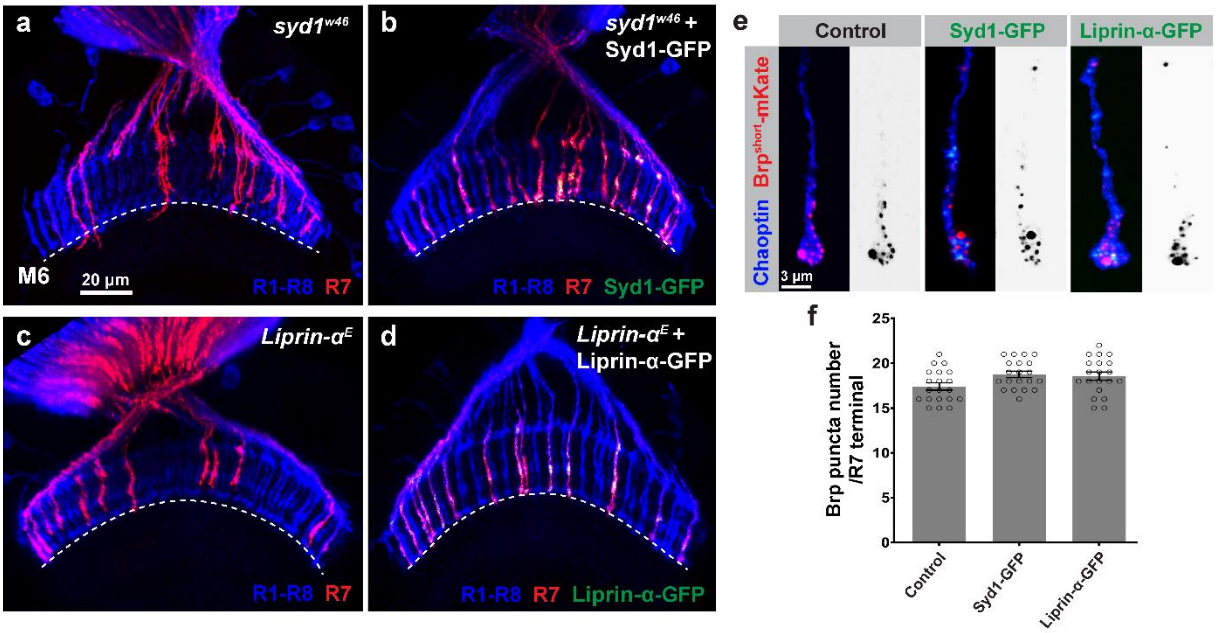

Supplementary Figure 12. Syd1 or Liprinα overexpression does not alter synapse number. a, *syd1* mutant R7 photoreceptors fails to target their correct medulla layer, M6. **b,** Syd1-GFP overexpression in *syd1* mutant R7 photoreceptors rescues mistargeting phenotypes. **c,** *liprina* mutant R7 photoreceptors fails to target their correct medulla layer, M6. **d,** Liprinα-GFP overexpression in *syd1* mutant R7 photoreceptors rescues mistargeting phenotypes. Repeated 2 times independently with similar results. **e,** Representative images of Brp^{short}-mKate labelled active zones in control, Syd1-GFP expressing and Liprinα-GFP expressing R7 axon terminals. Repeated 3 times independently with similar results. **f,** Number of Brp puncta in control, Syd1-GFP expressing and Liprinα-GFP expressing R7 axon terminals. n=20 terminals per condition. One-way ANOVA and Tukey HSD as post hoc test. Error bars denote mean ± SEM. No significant difference found between groups. Source data are provided as a Source Data file.

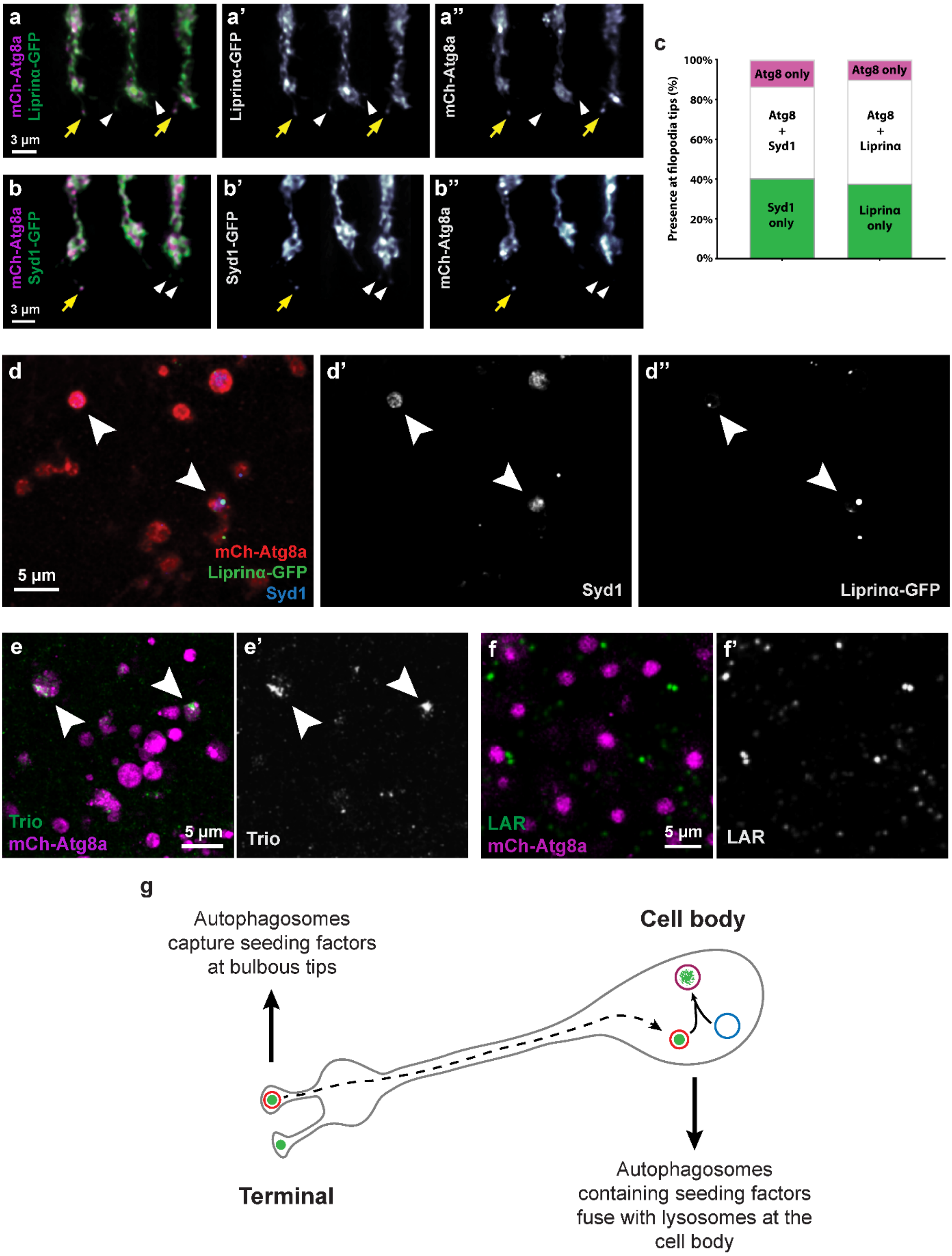

Supplementary Figure 13. Autophagosomes colocalize with synaptic seeding factors at filopodia tips and contain synaptic seeding factors in autolysosomes at cell bodies. a-a'', Representative R7 axon terminals expressing Liprin-α-GFP and mCherry-Atg8a. **b-b'',** Representative R7 axon terminals expressing Syd-1-GFP and mCherry-Atg8a. Yellow arrows: co-localization of Atg8a with synaptic seeding factors Liprin-α and Syd-1 at filopodia tips; white arrowheads: Liprin-α and Syd-1 at filopodia tips without apparent Atg8a co-

localization. Repeated 5-10 times independently with similar results. **c,** Percentages of Syd-1 only, Liprin-α only, Atg8a and Syd-1 together (Atg8a + Syd-1), Atg8a and Liprin-α together (Atg8a + Liprin-α), and Atg8a-only filopodia tips. n=30 terminals per condition. Note that most Atg8a-positive compartments are also positive for the synaptic seeding factors. All filopodia from 30 terminals were pooled for quantification. **d-d'',** Atg8a-positive autolysosomes contain Syd1 (detected with anti-Syd1 antibody) and Liprinα (UAS-Liprin-GFP) at photoreceptor cell bodies. **e-f',** Atg8a-positive autolysosomes at photoreceptor cell bodies contain Trio (**e-e'**) but not membrane receptor LAR (**f-f'**). Repeated 3 times independently with similar results. **g,** Schematic of proposed mechanism of degradation of synaptic seeding factors by autophagy in photoreceptor neurons, including capture at axon terminal filopodia tips and degradation during retrograde transport to the cell body, as first shown in vertebrate cell culture [1]. Source data are provided as a Source Data file.

Supplementary Tables

	Short lived (sF)		Long-lived (ℓF)	
	Life time	Number	Life time	Number
wild type	2.3 (1.6)	2.6 (1.8)	15 (10)	2.6 (1.4)
atg6	2.7 (1.9)	2 (2.3)	21 (16)	4.8 (1.2)
atg7	2.4 (1.6)	2.2 (1.9)	20 (15)	5.6 (2.6)
atg6, GMR>Atg6	2.2 (1.7)	2.9 (1.9)	13 (7)	1.4 (0.84)

Supplementary Table 1: Lifetimes (min) and average numbers of short- and long-lived filopodia at P60. Mean ± (standard deviation). Number distributions are shown in Supplementary Fig. 9.

	r3	r2B	E[f1]	r4	r5	Avg. bulbs
wild type	0.0122	0.0948	0.1291	0.0014	0.0108	1.653
atg6	0.0229	0.0932	0.2463	0.0025	0.0205	3.028
atg7	0.0189	0.1985	0.0955	0.0019	0.0170	2.501
atg6, GMR>Atg6	0.1032	0.1085	0.9515	0.1010	0.0018	1.644

Supplementary Table 2: Measured average rates of the data-driven model at P60.

The denotation is taken from the original model in Figure 3A of [2] and refer to the following filopodial transitions:

$$\text{Filopodia} \underset{r4}{\overset{r3=r2B*f1}{\leftrightarrows}} \text{Transient Bulbs} \overset{r5}{\rightarrow} \text{Stable Bulbs}$$

r3: measured rate of bulb formation, contains r2B * f1, unit: 1/min
r2B: propensity to form bulbs, cannot be measured, because feedback f1 reduces r2B, shown is the only possible fit of r2B, unit: 1/min
f1: negative feedback on bulb formation, cannot be measured, see r5, shown is the only possible fit of the data (r2B; smaller f1 indicates stronger feedback; f1=1 indicates no feedback
r4: measured rate of bulb disappearance, unit: 1/min
r5: measured rate of bulb stabilization, unit: 1/min
Avg. bulbs: average number of bulbs per time instance (min) over an hour (P60)
In blue: direct measurements

	$c_{1,sF}$	$c_{2,sF}$	$c_{1,\ell F}$	$c_{2,\ell F}$	c_3	c_4	c_5	c_6	B_{50}	$t_{1/2}$
wild type	1.82	0.43	0.28	0.07	0.024	1/120§	0.063	1/133	0.078	1000§
atg6	1.19	0.37	0.37	0.05	0.018	1/120§	0.068	1/133	0.716	1000§
atg7	1.48	0.42	0.45	0.05	0.033	1/120§	0.075	1/133	0.162	1000§
atg6, GMR>Atg6	2.13	0.45	0.17	0.08	0.033	0.071	0.001	1/133	3.733	1000§

Supplementary Table 3: Parameters of the data-driven model. All parameters in units min^{-1} except for B_{50} (unitless) and $t_{1/2}$ (min). §previously determined [2].

Supplementary References

1 Maday, S. & Holzbaur, E. L. Autophagosome biogenesis in primary neurons follows an ordered and spatially regulated pathway. *Dev Cell* **30**, 71-85, (2014).

2 Ozel, M. N. *et al.* Serial Synapse Formation through Filopodial Competition for Synaptic Seeding Factors. *Dev Cell*, (2019).
