# Supplementary material for: Autophagy-dependent filopodial kinetics restrict synaptic partner choice during Drosophila brain wiring
Source: Nat Commun. 2020 Mar 12;11:1325. doi: 10.1038/s41467-020-14781-4 (PMC7067798; doi:10.1038/s41467-020-14781-4)
Supplement: Supplementary file 3 — Reporting Summary [file 41467_2020_14781_MOESM3_ESM.pdf]

nature research

Corresponding author(s): P.Robin Hiesinger

Last updated by author(s): Jan 24, 2020

Reporting Summary

Nature Research wishes to improve the reproducibility of the work that we publish. This form provides structure for consistency and transparency in reporting. For further information on Nature Research policies, see Authors & Referees and the Editorial Policy Checklist .

Statistics

For all statistical analyses, confirm that the following items are present in the figure legend, table legend, main text, or Methods section.

n/a	Confirmed	
☐	☒	The exact sample size (*n*) for each experimental group/condition, given as a discrete number and unit of measurement
☐	☒	A statement on whether measurements were taken from distinct samples or whether the same sample was measured repeatedly
☐	☒	The statistical test(s) used AND whether they are one- or two-sided<br>*Only common tests should be described solely by name; describe more complex techniques in the Methods section.*
☒	☐	A description of all covariates tested
☐	☒	A description of any assumptions or corrections, such as tests of normality and adjustment for multiple comparisons
☐	☒	A full description of the statistical parameters including central tendency (e.g. means) or other basic estimates (e.g. regression coefficient) AND variation (e.g. standard deviation) or associated estimates of uncertainty (e.g. confidence intervals)
☒	☐	For null hypothesis testing, the test statistic (e.g. *F*, *t*, *r*) with confidence intervals, effect sizes, degrees of freedom and *P* value noted<br>*Give P values as exact values whenever suitable.*
☐	☒	For Bayesian analysis, information on the choice of priors and Markov chain Monte Carlo settings
☒	☐	For hierarchical and complex designs, identification of the appropriate level for tests and full reporting of outcomes
☐	☒	Estimates of effect sizes (e.g. Cohen's *d*, Pearson's *r*), indicating how they were calculated

Our web collection on statistics for biologists contains articles on many of the points above.

Software and code

Policy information about availability of computer code

Data collection	MATLAB codes for model parameter inference for model simulation are available through https://github.com/vkleist/Filo.
Data analysis	Standard statistical analysis, fully described in the Materials and Methods

For manuscripts utilizing custom algorithms or software that are central to the research but not yet described in published literature, software must be made available to editors/reviewers. We strongly encourage code deposition in a community repository (e.g. GitHub). See the Nature Research guidelines for submitting code & software for further information.

Data

Policy information about availability of data

All manuscripts must include a data availability statement. This statement should provide the following information, where applicable:

- Accession codes, unique identifiers, or web links for publicly available datasets
- A list of figures that have associated raw data
- A description of any restrictions on data availability

All datasets generated and/or analyzed during the study are available from the corresponding author on reasonable request.

Field-specific reporting

Please select the one below that is the best fit for your research. If you are not sure, read the appropriate sections before making your selection.

☒ Life sciences ☐ Behavioural & social sciences ☐ Ecological, evolutionary & environmental sciences

For a reference copy of the document with all sections, see nature.com/documents/nr-reporting-summary-flat.pdf

Life sciences study design

All studies must disclose on these points even when the disclosure is negative.

Sample size	Sample size details of all experiments are provided in figure legends. No statistical methods were used to predetermine sample size. For individual experiments, a sample size was chosen to reliably compare experimental groups.
Data exclusions	No data were excluded from the analysis
Replication	All experimental findings were reproduced at least twice. Information about how many times experiments were repeated with similar results are stated in figure legends.
Randomization	Samples were randomly allocated to experimental groups
Blinding	The investigators were not blinded to group allocations.

Reporting for specific materials, systems and methods

We require information from authors about some types of materials, experimental systems and methods used in many studies. Here, indicate whether each material, system or method listed is relevant to your study. If you are not sure if a list item applies to your research, read the appropriate section before selecting a response.

Materials & experimental systems

n/a	Involved in the study	
☐	☒	Antibodies
☒	☐	Eukaryotic cell lines
☒	☐	Palaeontology
☐	☒	Animals and other organisms
☒	☐	Human research participants
☒	☐	Clinical data

Methods

n/a	Involved in the study	
☒	☐	ChIP-seq
☒	☐	Flow cytometry
☒	☐	MRI-based neuroimaging

Antibodies

Antibodies used	The primary antibodies used in this study with given dilutions were as follows: mouse monoclonal anti-Chaoptin (1:200; Developmental Studies Hybridoma Bank); rat monoclonal anti-nCadherin (1:100; Developmental Studies Hybridoma Bank); rabbit monoclonal anti-Atg8 (1:100; Abcam); mouse monoclonal anti-Trio (1:50; Developmental Studies Hybridoma Bank); mouse monoclonal anti-LAR (1:50; Developmental Studies Hybridoma Bank); goat polyclonal anti-GFP (1:1000; Abcam); rat monoclonal anti-GFP (1:500; BioLegend); rabbit polyclonal anti-CD4 (1:600; Atlas Antibodies); rabbit polyclonal anti-DsRed (1:500; ClonTech); rabbit anti-Syd1 (1:500; gift from Sigrist Lab). The secondary antibodies Cy3, Cy5 (Jackson ImmunoResearch Laboratories) and Alexa488 (Invitrogen) were used in 1:500 dilution.
Validation	See details in the Results section of this manuscript and original publications of these antibodies

Animals and other organisms

Policy information about studies involving animals; ARRIVE guidelines recommended for reporting animal research

Laboratory animals	Drosophila melanogaster
Wild animals	n/a
Field-collected samples	n/a
Ethics oversight	No ethical approval or guidance is required for studies using Drosophila melanogaster as a model organism.

Note that full information on the approval of the study protocol must also be provided in the manuscript.

October 2018
