# Supplementary material for: Autophagy-dependent filopodial kinetics restrict synaptic partner choice during Drosophila brain wiring
Source: Nat Commun. 2020 Mar 12;11:1325. doi: 10.1038/s41467-020-14781-4 (PMC7067798; doi:10.1038/s41467-020-14781-4)
Supplement: Supplementary file 4 — Description of Additional Supplementary Files [file 41467_2020_14781_MOESM4_ESM.docx]

**Description of Additional Supplementary Files**

Supplementary Movie 1
Ectopic synapses at autophagy-deficient R7 terminals are stable

Supplementary Movie 2
Autophagosome formation destabilizes bulbous tip filopodia.

Supplementary Movie 3
Autophagy regulates filopodial dynamics at developing axon terminals
