# Supplementary material for: Autophagy-dependent filopodial kinetics restrict synaptic partner choice during Drosophila brain wiring
Source: Nat Commun. 2020 Mar 12;11:1325. doi: 10.1038/s41467-020-14781-4 (PMC7067798; doi:10.1038/s41467-020-14781-4)
Supplement: Supplementary file 2 — Peer Review [file 41467_2020_14781_MOESM2_ESM.pdf]

Reviewers' comments:

Reviewer #1 (Remarks to the Author):

In this manuscript, Kiral and colleagues investigate the role of autophagy in brain development using Drosophila as a model system. More specifically, the authors address the question of whether and how autophagy contributes to synapse formation at the level of filopodial dynamics in the developing fly brain. They found that mutants bearing Atg7 or Atg6 deficient photoreceptor neurons exhibit normal eye morphology. However, they show markedly increased "on" transient amplitudes and decreased "stripe deviation", which are indicative of increased neurotransmission and visual attention behavior, respectively. Transgenic expression of atg6 specifically in photoreceptor neurons rescued the above mentioned mutant phenotypes. Furthermore, the number of synapses was significantly increased in autophagy deficient photoreceptors. Loss of autophagy in R7 photoreceptors, in particular, increased the number of their inappropriate postsynaptic partners. Using the GFP reconstitution across synaptic partners (GRASP) method, the authors found that the synapses formed between the autophagy deficient R7 neurons and Mi1, C2 and Mi4 incorrect partners are functional. Further analysis showed that autophagosomes form in synaptogenic filopodia of R7 axon terminals followed by filopodia collapse in wild type flies. Notably, wild type axon terminals formed 1-2 synaptogenic filopodia. By contrast, autophagy depleted R7 photoreceptors exhibited increased number of synaptogenic filopodia in their axon terminals at any time point, suggesting that bulbous tip filopodia are stabilized when autophagy is impaired. Moreover, the synaptic seeding factors Syd-1 and Liprin-ɑ were found to colocalize with Atg-8-positive foci in wild-type R7 axon terminals, suggesting that autophagy controls their availability.

This is an interesting paper, which suggests a role for autophagy in the regulation of filopodia dynamics at developing axon terminals, contributing to wiring specificity. A previous study by the main author (Ref.29) showed that autophagosomes form at the axon terminals of developing photoreceptor neurons R1-R6. In fact, this is a follow up study that sheds light on the function of autophagy in developing Drosophila brains.

However, the idea that autophagy sets a threshold for kinetic restriction across the R7 axon terminal so as to exclude inappropriate postsynaptic partners from forming synapses with this photoreceptor needs further validation. What might be the context-dependent molecular interactions that drive such a specificity as the authors assume in the Discussion section? The authors should elaborate more this suggestion. Moreover, what determines the spatial availability of appropriate synaptic partners?

The finding that autophagy-deficient flies have increased neurotransmission and increased visual attention (Figure 1) is intriguing. Do the authors suggest that autophagy is detrimental for vision? Does autophagy have any beneficial role (possibly in preventing the degeneration of photoreceptor cells which is normally observed along ageing) which can counterbalance the predicted evolutionary disadvantage of wild-type flies?

Figure 3 shows that the R7 photoreceptor cells in autophagy-deficient flies form aberrant connections with neuronal cell types they don't normally connect to. Do these new connections have any functional consequences for the behavior of flies? Do atg7 or atg6 mutant flies have any defects in "motion-detection" associated response (which is regulated by Mi1 and Mi4 neurons)? I believe the authors need to strengthen the finding that the supernumerary synapses are functional using some behavioral assays (apart from GRASP data shown in figure 4).

The authors propose that autophagy modulates filopodia dynamics and synapse formation by directly controlling the availability of synaptic seeding factors. However, the numbers of measured synaptogenic filopodia in wild type and autophagy deficient R7 photoreceptors display a high level of variability as evidenced in Fig. 6h (please see, error bars). Did the authors examine whether impaired autophagy in R7 axon terminals somehow affects the Lar and Trio components of the Lar- Liprin-ɑ –

Syd1-Trio pathway, in addition to the synaptic seeding factors Syd1 and Liprin-α?

The authors showed that the autophagosomal marker Atg8::mCherry colocalizes with Syd1::GFP and Liprin-α::GFP at filopodia tips. In addition, they observed Atg8-positive vehicles containing the Syd1 synaptic seeding factor. Did they also observe Liprin-α to be enclosed by Atg8-positive structures?

Is there evidence that Atg8 physically interacts with Liprin-α and Syd1? (for example, based on immunoprecipitation experiments or proteomics analysis).

To what degree are the findings of this study relevant to more complex organisms? Are the described mechanisms mostly specific to Drosophila? This should be discussed by the authors.

Reviewer #2 (Remarks to the Author):

The manuscript by Kiral et al. focuses on the role of autophagy in regulating filopodial dynamics and synaptic partner choice during circuit development in the Drosophila visual system. Using a powerful combination of sophisticated genetics, live imaging, circuit mapping, behavioral analyses, and mathematical modeling, the authors provide compelling evidence that autophagy normally limits the formation of synaptogenic filopodia thereby restraining the number of synaptic contacts made by a growth cone. They further argue that autophagy inhibits synaptogenesis via non-selective degradation of synaptic seeding factors. This is an exciting and novel hypothesis as it proposes that autophagy regulates synaptic specificity (merely) by regulating the tempo of the transition of exploratory growth cone to synaptogenic filopodium. How growth cones undergo this crucial transition is not at all clear, making this study of great interest to developmental neurobiologists.

The manuscript is logically organized and clearly presented. Overall, the data are of very high quality. The cross talk between the experimental and modeling approaches is excellent and argues that the observed dynamic changes are sufficient to explain the later synaptic differences. The evidence presented that loss of atg6 or 7 leads to ectopic synaptic contacts that result from an increased number of bulbous/synaptogenic filopodia is strong.

My only moderate concern lies in the approaches undertaken to investigate the relative localization of the autophagy machinery and synaptic seeding factors. All of the tools used to probe these questions are overexpressed transgenes (Atg5-GFP, Atg8-GFP, Syd-1-GFP, Liprinα-GFP). Several issues regarding these reagents should be addressed.
(1) Are these transgenes functional? (Do they rescue LOF phenotypes?)
(2) How highly are they overexpressed? This is important here because if they are expressed at levels well over wild-type, they may be overwhelming the system and thus not reflecting localization of the endogenous proteins.
(3) It seems particularly critical to know whether their overexpression changes synapse formation. For example, does overexpression of either of the synaptic seeding factors lead to increased synapse formation? This might be expected if (as the authors argue) levels of these proteins in filopodia are limiting and control which of the filopodia is destined to be synaptogenic. On the other hand, does overexpression of the autophagy proteins lead to decreased synapse formation? This seems logical given the data presented for atg6 overexpression. If altering the levels of these proteins does not change synapse number/targeting, the authors may wish to modify the conclusions of this set of experiments.

Minor concerns.
It is not clear to this reviewer why the quantified stripe deviation data for atg6, GMR>atg6 flies (Figure 1 I) resemble that of controls given that the traces/heat maps (Figure 1 H) seem different between the two genotypes. It appears that the atg6 rescue animals deviate from the straight line

more than controls.

The trans-Tango experiment to map the ectopic synapses formed by R7 in the atg6 mutant is basically convincing. The authors quantify the inappropriate cell types contacted by R7 in this background. With respect to the control, the authors state in the text that trans-Tango with wild-type R7s “mainly labeled” Dm8s and Tm5s. Can this be quantified? This would help to judge the accuracy of the trans-Tango method in this system.

In the Discussion on line 332-333, the authors speculate that “different nenal thresholds for kinetic restrictions…”, which does not make sense.

Reviewer #3 (Remarks to the Author):

This manuscript by Kiral and colleagues introduces a reasonably supported model of how autophagic protein degradation controls synaptogenesis in the optic lobe of the brain of the popular animal model Drosophila. Loss of autophagy is found to increase the number of synaptogenic filopodia likely via increased stability of synaptogenic proteins. Vice versa, genetically promoted autophagy is thought to decrease filopodia and synapse numbers. Importantly, their data support that autophagy acts on a global level by setting the threshold for synaptogenesis, rather than being specifically induced in incorrect synapses. I think this work is very important for a wide audience, so I support publication if a number of existing issues are addressed properly.

Major comments

1. Fig. S1: unpaired t test is inappropriate for comparing multiple genotypes (4 in this case, 1 being the same control for the other 3). For multiple comparisons, a different test such as ANOVA or Kruskal-Wallis needs to be used depending whether data distribution is normal (that is, Gaussian) or non-normal, respectively. I suspect that for Atg6 mutants, it is non-normal because there are hardly any autophagosomes. Similar issues should be dealt with in case of Figs 2e-g, 5g, 6j-k. Please update all relevant p values accordingly.

2. Trans-TANGO and GRASP labeling experiments are only done for Atg6 mutants, which might be a problem as Atg6 acts not only in the autophagy but also in endocytic and biosynthetic lysosomal protein transport pathways. I thus suggest to test another autophagy mutant if it is feasible. If these experiments are not possible for Atg7 mutants, please see alternative scenarios in comment 3.

3. Strictly speaking, no genetic rescue experiments have been performed because Atg6 overexpression led to a gain-of-function phenotype. A real rescue experiment (eg. using genomic promoter-driven Atg6) would therefore be a welcome addition, or if that is not feasible, one more Atg gene mutant or knockdown should be analyzed at least in some experiments. Drosophila null mutants have been published for eg. Atg1, FIP200, Atg13, Atg101, Atg14, Atg9, Atg5, Atg16, and several RNAi lines have been successfully used in neurons, so plenty of suitable reagents are available for this.

Minor comments

4. The authors may want to demonstrate that the Atg6 overexpression effect is autophagy-specific (see comment 2 for its non-autophagic roles). Simply Atg6 overexpression effect needs to be analyzed on an Atg7 mutant (or some Atg RNAi) background.

5. There are two paralogs of Atg8 in Drosophila: Atg8a that is expressed in all cells and Atg8b whose expression is practically restricted to the testis based on high-throughput data. Please specify throughout the text and figures which Atg8 protein/gene is being looked at.

6. A brief description of how trans-Tango labeling works would make it easier to understand those experiments.

7. Fig S3. Only N-terminally tagged Atg8 reporters are functional as the C-terminus is cleaved by Atg4 to expose the Glycine residue that is conjugated to the lipid PE. Thus, please use GFP-Atg8(a?) instead of Atg8-GFP and specify which paralog (Atg8a vs Atg8a) it is. The same goes for Fig S7: Atg8-mCh should be corrected (mCherry-Atg8a?)

8. Line 268, "large Atg8-positive multivesicular bodies containing Syd-1" The true identity of the speculated multivesicular bodies is questionable - these may as well be autolysosomes. Please refrain from claiming this about the perinuclear Atg8a-positive vesicles, unless it is supported by eg. TEM.

9. Line 332 what is "nenal"?

Revision Plan for Kiral et al., NCOMMS-19-30077

Response to Reviewers

We would like to thank all three reviewers for their thoughtful comments on the manuscript, their positive assessments as well as excellent suggestions for improvement. We agreed with almost all comments and suggestions and have adopted them as described below. We especially agreed with the experimental suggestions to the extent that they were technically feasible. For the revised manuscript, we have therefore performed several additional experiments that were added as panels to main Figures 1-4 as well as in six additional Supplemental Figures and several additions to the existing Supplemental Figures. Specifically, we have performed a full set of experiments (neurotransmission in ERG recordings, Brp synapse counts, filopodia characterization, trans-Tango and activity-GRASP) for another independent autophagy mutant, *atg18*, which consistently behaved as a hypomorph for autophagy function. These experiments, plus two additional autophagy RNAi knock-downs (*atg5* and *atg16*) strongly support the findings made with the *atg6* and *atg7* mutants. In addition, we have added a new behavioral assay showing that basic optomotor behavior is unaffected in flies lacking autophagy in their photoreceptors. We also now show autophagosomes engulfing a third cytosolic protein (Trio), but not the membrane receptor Lar. Finally, important control experiments validated the use of GFP-tagged reporter constructs and the trans-Tango method. We are glad to report that these experimental additions, together with textual revisions as requested by the reviewers, consistently strengthened our findings on the role of developmental autophagy in synapse formation. To facilitate the review of our revision efforts, the *reviewer comments are shown below in full in blue*, followed by our detailed responses.

Reviewers' comments:

Reviewer #1: *In this manuscript, Kiral and colleagues investigate the role of autophagy in brain development using Drosophila as a model system. More specifically, the authors address the question of whether and how autophagy contributes to synapse formation at the level of filopodial dynamics in the developing fly brain. They found that mutants bearing Atg7 or Atg6 deficient photoreceptor neurons exhibit normal eye morphology. However, they show markedly increased "on" transient amplitudes and decreased "stripe deviation", which are indicative of increased neurotransmission and visual attention behavior, respectively. Transgenic expression of atg6 specifically in photoreceptor neurons rescued the above mentioned mutant phenotypes. Furthermore, the number of synapses was significantly increased in autophagy deficient photoreceptors. Loss of autophagy in R7 photoreceptors, in particular, increased the number of their inappropriate postsynaptic partners. Using the GFP reconstitution across synaptic partners (GRASP) method, the authors found that the synapses formed between the autophagy deficient R7 neurons and Mi1, C2 and Mi4 incorrect partners are functional. Further analysis showed that autophagosomes form in synaptogenic filopodia of R7 axon terminals followed by filopodia collapse in wild type flies. Notably, wild type axon terminals formed 1-2 synaptogenic filopodia. By contrast, autophagy depleted R7 photoreceptors exhibited increased number of synaptogenic filopodia in their axon terminals at any time point, suggesting that bulbous tip filopodia are stabilized when autophagy is impaired. Moreover, the synaptic seeding factors Syd-1 and Liprin-α were found to colocalize with Atg-8-positive foci in wild-type R7 axon terminals, suggesting that autophagy controls their availability.*

This is an interesting paper, which suggests a role for autophagy in the regulation of filopodia dynamics at developing axon terminals, contributing to wiring specificity. A previous study by the main author (Ref.29) showed that autophagosomes form at the axon terminals of developing photoreceptor neurons R1-R6. In fact, this is a follow up study that sheds light on the function of autophagy in developing Drosophila brains.

However, the idea that autophagy sets a threshold for kinetic restriction across the R7 axon terminal so as to exclude inappropriate postsynaptic partners from forming synapses with this photoreceptor needs further validation. What might be the context-dependent molecular interactions that drive such a specificity as the authors assume in the Discussion section? The authors should elaborate more this suggestion. Moreover, what determines the spatial availability of appropriate synaptic partners?

Response 1.1: These are important questions and we have taken this opportunity to elaborate in the Discussion section as requested, especially on the question: how does a ubiquitous mechanism through alteration of a kinetic threshold contribute to synaptic specificity? In short, we posit that specificity can emerge through the composite action of several factors that by themselves appear 'permissive', e.g. positioning and kinetics. This mechanism operates in addition to attractive and repellent molecular interactions, that are more commonly studied in this context. Remarkably, we found that those interneurons whose dendrites are in the same layer as autophagy-deficient R7 axon terminal filopodia formed more synapses than those interneurons with dendrites in adjacent layers. It is likely that the spatial availability of each of these interneurons is under their own specific developmental regulation and independent from the R7 axons until the time of synapse formation. Given that for at least six incorrect synaptic partnerships functional synapses are formed supports a model whereby restrictions to their 'meeting each other' contribute to specificity. The idea of a threshold for kinetic restriction across the R7 terminal is based on the observation that the same kinetic, filopodial and synaptic alterations occur across the entire terminal, from M1-M6 (Fig. 7). We have clarified each of these points where applicable in both the Results and Discussion sections.

The finding that autophagy-deficient flies have increased neurotransmission and increased visual attention (Figure 1) is intriguing. Do the authors suggest that autophagy is detrimental for vision? Does autophagy have any beneficial role (possibly in preventing the degeneration of photoreceptor cells which is normally observed along ageing) which can counterbalance the predicted evolutionary disadvantage of wild-type flies?

Response 1.2: This is a very interesting idea. Loss of autophagy is indeed well known to lead to neurodegeneration in fly photoreceptors as in many other neurons. We and others have investigated the effect of modulating levels of autophagy, but have found no protective effect for overexpression in fly photoreceptors. To explore the effects of reduced autophagy on vision in more detail, we have performed a newly established optomotor assay and evaluated more parameters of the Buridan assay, as described in detail in the following Response 1.3. In brief, optomotor behavior is unaffected, arguing against a simple detrimental role of autophagy for basic vision. However, the synaptic alterations caused by loss of developmental autophagy in photoreceptors do cause complex behavioral changes to a visual stimulus, as shown for additional parameters in the Buridan's paradigm (center deviation and distance walked). The new behavioral data are added in the new Supplemental Figure S4 and discussed in Response 1.3 in detail below. We think that the developmental role of autophagy may be separate from its role during adult maintenance and have added this point in a new paragraph in the Discussion in the revised manuscript.

Figure 3 shows that the R7 photoreceptor cells in autophagy-deficient flies form aberrant connections with neuronal cell types they don't normally connect to. Do these new connections have any functional consequences for the behavior of flies? Do atg7 or atg6 mutant flies have any defects in "motion-detection" associated response (which is regulated by Mi1 and Mi4 neurons)? I believe the authors need to strengthen the finding that the supernumerary synapses are functional using some behavioral assays (apart from GRASP data shown in figure 4).

Response 1.3: We have followed the reviewer's suggestion and further investigated the outcome of the supernumerary synapses in behavior experiments. Specifically, we have performed an

optomotor assay with tethered, flying flies in a virtual flight arena. Loss of autophagy in photoreceptors did not significantly affect the ability of flies to follow counter-clockwise and clockwise motion. This finding suggests that optomotor behavior is sufficiently robust to not be altered by a minority of additional, incorrect synaptic connections as long as all correct connections remain. In addition, we have evaluated additional behavioral parameters in the Buridan's paradigm (also in response to Reviewer 2, see Response 2.4). Flies can see the visual stimuli in this assay as well, but walk more between the two stimuli (black stripes), with less deviation in any direction. Overexpression of *atg6* overcompensates this effect and causes flies to walk more away from the center. We conclude that supernumerary synapses do affect more complex behavior, while basic vision, including optomotor behavior, remain intact. All additional data are presented in the new Supplemental Figure S4 and described in the revised Results section.

The authors propose that autophagy modulates filopodia dynamics and synapse formation by directly controlling the availability of synaptic seeding factors. However, the numbers of measured synaptogenic filopodia in wild type and autophagy deficient R7 photoreceptors display a high level of variability as evidenced in Fig. 6h (please see, error bars). Did the authors examine whether impaired autophagy in R7 axon terminals somehow affects the Lar and Trio components of the Lar- Liprin-α – Syd1-Trio pathway, in addition to the synaptic seeding factors Syd1 and Liprin-α?

Response 1.4: Yes, we do indeed observe increased variability with increasing numbers of synaptogenic filopodia in autophagy-deficient axon terminals. We also think that autophagy may affect filopodia differentially, depending on their size, lifetime and the proteins present. For the revision, we followed the reviewer's suggestion and experimentally tested whether Lar and Trio are affected similar to synaptic seeding factors, but encountered technical feasibility issues. For Liprin-GFP and GFP-Syd-1 we could limit their expression to a single cell marked with a membrane-tagged GFP, because neither causes obvious overexpression defects (Ozel et al., 2019). In contrast, Lar and Trio overexpression both cause known overexpression defects. We therefore performed a series of immunohistochemistry experiments using antibodies against Lar and Trio. The problem with antibody labeling in dense brain neuropil is that immunoreactivity cannot safely be assigned to a single filopodium. We therefore performed control antibody labeling experiments in which single, GFP-marked axon terminals were mutant for either *lar* or *trio*. However, Lar and Trio did not show a quantifiable reduction in the regions of the mutant axon terminals marked by membrane GFP (see Figure for Reviewers at the end of this document). We validated that both mutants and antibodies worked. However, based on EM we know that the GFP-marked region of an R7 axon terminal contains hundreds of unmarked and likely Lar or Trio-positive axonal and dendritic processes from many other cells. Our results suggest that in the case of Lar and Trio too many Lar- and Trio-positive other neuronal processes prevent the observation of a quantifiable reduction within the GFP-marked axon terminal areas. However, we could use the antibodies to identify Trio and Lar in autophagosomes in cell bodies, as described below.

The proposed mechanism of filopodial destabilization may not require a specific cargo protein, since triggering autophagy in the small space of a filopodial tip could easily destabilize the filopodium even if cargo degradation were rather unspecific. We used the validated Lar and Trio antibodies to test whether Lar or Trio can be found inside large Atg8-positive compartments in the photoreceptor cell bodies. Similar to Syd-1 and Liprin-alpha, Trio was present in Atg8-positive compartments (new Suppl. Fig. S13e-f). However, we did not find Lar inside Atg8-positive compartments, suggesting that cytoplasmic proteins are more easily engulfed than the Lar membrane receptor. We have added these data to the Results section and strengthened the corresponding Discussion point on cargo specificity.

The authors showed that the autophagosomal marker Atg8::mCherry colocalizes with Syd1::GFP and Liprin-α::GFP at filopodia tips. In addition, they observed Atg8-positive vehicles containing the Syd1

synaptic seeding factor. Did they also observe Liprin-α to be enclosed by Atg8-positive structures?

Response 1.5: We have performed additional experiments using *Liprin-α::GFP* (a *Drosophila* Liprin antibody is not available and tests of a mammalian antibody did not validate). We co-expressed mCherry-Atg8a and Liprin-α-GFP with GMR-Gal4 and then used the antibody against Syd-1 to test for colocalization. The result is presented in Suppl. Fig. S13d-d''. Indeed, in most cases, Liprin-α and Syd-1 are present in the same autolysosome . This finding supports the idea that both seeding factors are subject to autophagic degradation during development.

Is there evidence that Atg8 physically interacts with Liprin-α and Syd1? (for example, based on immunoprecipitation experiments or proteomics analysis).

Response 1.6: We are not aware of such evidence. However, as mentioned in Response 1.4, the proposed mechanism does not require cargo-specificity and may engulf other proteins, including Trio (new Suppl. Fig. S13e-f).

To what degree are the findings of this study relevant to more complex organisms? Are the described mechanisms mostly specific to Drosophila? This should be discussed by the authors.

Response 1.7: We think it highly likely that filopodial dynamics play similar roles in all developing brains. However, long-term observation of filopodial dynamics in intact developing brains has been very challenging in all model systems. However, a previous observation of increased spine numbers in autophagy-deficient neurons in mice may in part be explained by our findings (Tang et al., 2014). We added a discussion of the potential significance of our findings in the revised Discussion section.

Reviewer #2: *The manuscript by Kiral et al. focuses on the role of autophagy in regulating filopodial dynamics and synaptic partner choice during circuit development in the Drosophila visual system. Using a powerful combination of sophisticated genetics, live imaging, circuit mapping, behavioral analyses, and mathematical modeling, the authors provide compelling evidence that autophagy normally limits the formation of synaptogenic filopodia thereby restraining the number of synaptic contacts made by a growth cone. They further argue that autophagy inhibits synaptogenesis via non-selective degradation of synaptic seeding factors. This is an exciting and novel hypothesis as it proposes that autophagy regulates synaptic specificity (merely) by regulating the tempo of the transition of exploratory growth cone to synaptogenic filopodium. How growth cones undergo this crucial transition is not at all clear, making this study of great interest to developmental neurobiologists.*

The manuscript is logically organized and clearly presented. Overall, the data are of very high quality. The cross talk between the experimental and modeling approaches is excellent and argues that the observed dynamic changes are sufficient to explain the later synaptic differences. The evidence presented that loss of atg6 or 7 leads to ectopic synaptic contacts that result from an increased number of bulbous/synaptogenic filopodia is strong.

My only moderate concern lies in the approaches undertaken to investigate the relative localization of the autophagy machinery and synaptic seeding factors. All of the tools used to probe these questions are overexpressed transgenes (Atg5-GFP, Atg8-GFP, Syd-1-GFP, Liprin-α-GFP). Several issues regarding these reagents should be addressed.
(1) Are these transgenes functional? (Do they rescue LOF phenotypes?)

Reponse 2.1: We completely agree and were careful to only use GFP-tagged proteins that are known, previously used reporters and have performed additional validation experiments to address these

concerns. The Syd-1 and Liprin-α transgenes have been tested in rescue experiments by us and others in several contexts (Özel et al., 2019 Dev Cell; Li, Tian et al., 2014, J. Neurosci). GFP-Atg5 and GFP-Atg8a are very widely used transgenes to assay the progression of autophagosome maturation (e.g. Erika Holzbaur's paper Maday and Holzbaur 2012, 2014). We see the same orderly sequence of recruitment in live imaging, indicating autophagosome formation as published. In addition, we performed electroretinogram (ERG) recordings and synapse number analysis for GFP-Atg5 and GFP-Atg8a overexpression (new Suppl. Fig. S7) as well as Syd1-GFP and Liprin-α-GFP overexpression (new Suppl. Fig. S12). All validation experiments were successful: none of the overexpressed reporters significantly changed synapse numbers or ERG on-transient and depolarization. We further rescued the syd-1 and liprin-α mutants with these overexpression constructs (new Suppl. Fig. S12). Based on these experiments and the established and published work with these reporters, we are confident that these four reporters provide accurate read-outs.

(2) How highly are they overexpressed? This is important here because if they are expressed at levels well over wild-type, they may be overwhelming the system and thus not reflecting localization of the endogenous proteins.

Response 2.2: The question regarding overexpression is indeed very important. For Syd-1 and Liprin we have extensively tested the effect of overexpression on filopodial dynamics and their accumulation in synaptogenic filopodia in Ozel et al. (2019, Dev. Cell). Here we showed that overexpressed Syd1-GFP or Liprin-alpha-GFP does not alter filopodial dynamics or even their accumulation in synaptogenic filopodia. GFP-Atg5 and GFP-Atg8a are only used as reporters for autophagosomes in filopodia. Previous reports indicated no significant induction of autophagy by these two proteins. As described in Response 2.1, overexpression of GFP-Atg5 and GFP-Atg8a with GMR-Gal4 in this system did not affect any of the measured read-outs (Suppl. Fig. S7).

(3) It seems particularly critical to know whether their overexpression changes synapse formation. For example, does overexpression of either of the synaptic seeding factors lead to increased synapse formation? This might be expected if (as the authors argue) levels of these proteins in filopodia are limiting and control which of the filopodia is destined to be synaptogenic. On the other hand, does overexpression of the autophagy proteins lead to decreased synapse formation? This seems logical given the data presented for atg6 overexpression. If altering the levels of these proteins does not change synapse number/targeting, the authors may wish to modify the conclusions of this set of experiments.

Response 2.3: As mentioned in Response 2.2, published experiments do not indicate that Atg5 or Atg8 overexpression induce autophagy, in contrast to Atg6 (Beclin1) overexpression, which is known to induce autophagy and confirmed to do so in the manuscript (and not able to do so in the background of an atg7 mutant, see Response 3.4. As described in Response 2.1 and Response 2.2, overexpression of GFP-Atg5 and GFP-Atg8a did not affect neurotransmission or synapse numbers (see details above and new Suppl. Fig. S7).

Minor concerns.
It is not clear to this reviewer why the quantified stripe deviation data for atg6, GMR>atg6 flies (Figure 1 I) resemble that of controls given that the traces/heat maps (Figure 1 H) seem different between the two genotypes. It appears that the atg6 rescue animals deviate from the straight line more than controls.

Response 2.4: This is correct. The stripe deviation parameter only captures walking deviations from the line between the two stimuli (black stripes). Overexpression of atg6 rescues strip deviation as well as other parameters, e.g. center deviation (new Suppl. Fig. S4a,b). However, overexpression of atg6 also leads to more walking away from the line between the stimuli (new Suppl. Fig. S4a-d): they

walk more along the circumference of the arena, which is apparent in Fig. 1h and Fig S4a,b. These behavioral changes are consistent with the overcompensation of atg6 in rescue experiments for all other assays. See also Response 1.3. We have added this data and explanation to the corresponding Results section.

The trans-Tango experiment to map the ectopic synapses formed by R7 in the atg6 mutant is basically convincing. The authors quantify the inappropriate cell types contacted by R7 in this background. With respect to the control, the authors state in the text that trans-Tango with wild-type R7s "mainly labeled" Dm8s and Tm5s. Can this be quantified? This would help to judge the accuracy of the trans-Tango method in this system.

Response 2.5: This is correct - Dm8 and Tm5 are indeed the most labeled cells in the trans-Tango experiments. In order to see single (or very few) of these cells, we applied a very sparse labeling protocol and show a representative example of this in the new Suppl. Fig. S6. In very sparse labelings, almost only these two cell types are visible, supporting the specificity of trans-Tango using the yR7-specific Rh4-driver. However, these data cannot easily be quantified. We have added the data and reference to the new Suppl. Fig. 6 in the trans-Tango Results section.

In the Discussion on line 332-333, the authors speculate that "different nenal thresholds for kinetic restrictions…", which does not make sense.

Response 2.6: 'neuronal', corrected with our apologies!

Reviewer #3: *This manuscript by Kiral and colleagues introduces a reasonably supported model of how autophagic protein degradation controls synaptogenesis in the optic lobe of the brain of the popular animal model Drosophila. Loss of autophagy is found to increase the number of synaptogenic filopodia likely via increased stability of synaptogenic proteins. Vice versa, genetically promoted autophagy is thought to decrease filopodia and synapse numbers. Importantly, their data support that autophagy acts on a global level by setting the threshold for synaptogenesis, rather than being specifically induced in incorrect synapses. I think this work is very important for a wide audience, so I support publication if a number of existing issues are addressed properly.*

Major comments

1. Fig. S1: unpaired t test is inappropriate for comparing multiple genotypes (4 in this case, 1 being the same control for the other 3). For multiple comparisons, a different test such as ANOVA or Kruskal-Wallis needs to be used depending whether data distribution is normal (that is, Gaussian) or non-normal, respectively. I suspect that for Atg6 mutants, it is non-normal because there are hardly any autophagosomes. Similar issues should be dealt with in case of Figs 2e-g, 5g, 6j-k. Please update all relevant p values accordingly.

Response 3.1: We have corrected the statistical tests as suggested by the reviewer. We first performed a normality test to check if all groups are normally distributed in a given data. If so, we performed parametric one-way ANOVA; if not, we performed nonparametric Kruskal-Wallis test. The new tests support all previous conclusions. The new test are described in the Results section and in the Materials and Methods.

2. Trans-TANGO and GRASP labeling experiments are only done for Atg6 mutants, which might be a problem as Atg6 acts not only in the autophagy but also in endocytic and biosynthetic lysosomal protein transport pathways. I thus suggest to test another autophagy mutant if it is feasible. If these

experiments are not possible for Atg7 mutants, please see alternative scenarios in comment 3.

<u>Response 3.2:</u> We followed the reviewer's advice and performed a full set of experiments for another, independent autophagy mutant. The *atg7* mutant chromosome is not easily adaptable for available Trans-Tango and GRASP reagents; we therefore identified *atg18*, which is required for PI3P (Phosphatidylinositol 3-phosphate) recruitment to the phagophore and LC3 (Atg8) lipidation. The *atg18* null mutant behaved consistently as a hypomorph for autophagy. Loss of atg18 in mutant clones reveals a significant, but (in contrast to *atg6* and *atg7*) not complete loss of Atg8-positive compartments (new data in Suppl. Fig. S1). Consequently, loss of atg18 in photoreceptors leads to increased neurotransmissions (new data in Fig. 1c-e), and increased Brp-positive synapses (new data in Fig. 2). Trans-Tango revealed increased numbers of postsynaptic cells at intermediate levels (new data in Fig. 3c, d) and a milder, but significant increase of activity-dependent GRASP signal (new data in Fig. 4g-i). Finally, loss of atg18 leads to increased numbers of synaptogenic filopodia (new data in Suppl. Fig. S10), providing a complete set of experimental evidence for the effects seen in atg6 and atg7 for another autophagy mutant.

3. Strictly speaking, no genetic rescue experiments have been performed because Atg6 overexpression led to a gain-of-function phenotype. A real rescue experiment (eg. using genomic promoter-driven Atg6) would therefore be a welcome addition, or if that is not feasible, one more Atg gene mutant or knockdown should be analyzed at least in some experiments. Drosophila null mutants have been published for eg. Atg1, FIP200, Atg13, Atg101, Atg14, Atg9, Atg5, Atg16, and several RNAi lines have been successfully used in neurons, so plenty of suitable reagents are available for this.

<u>Response 3.3:</u> It was very difficult in our hands to hit the exactly right level of *atg6* overexpression to precisely achieve endogenous *atg6* levels and a genomic *atg6* rescue construct has never been generated. We attempted another set of experiments at 18C and 25C, but this did not significantly alter the levels of rescue through *atg6* overexpression. In all our experiments, *atg6* overexpression consistently overcompensated for *atg6* loss with the phenotypes expected for mild gain-of-function. In addition, *atg6* overexpression had no effect if autophagy was disrupted through loss of the core component *atg7* (see also <u>Response 3.4</u> below). We nonetheless followed the reviewers advice to analyze more *atg* mutants. A full set of experiments for *atg18* provided highly consistent data for an autophagy hypomorph (see <u>Response 3.2</u> and new data in Figs. 1,2,3,4,S1,S10). In addition, we performed RNAi knock-down experiments for *atg5* and *atg16*, which both also yielded increased neurotransmission (ERG on-transients) and Brp synapse counts (new Suppl. Fig. S2). Together, we now provide data for five independent autophagy mutants or knock-downs for the neurotransmission and synapse counts.

Minor comments

4. The authors may want to demonstrate that the Atg6 overexpression effect is autophagy-specific (see comment 2 for its non-autophagic roles). Simply Atg6 overexpression effect needs to be analyzed on an Atg7 mutant (or some Atg RNAi) background.

<u>Response 3.4:</u> We performed this excellent experimental suggestion and overexpressed *atg6* in *atg7* mutant photoreceptors. Remarkably, *atg6* overexpression did not have any effect in an *atg7* mutant background, i.e. the *atg7;GMR>atg6* synapse numbers and synaptogenic filopodia phenotype was indistinguishable from the *atg7* mutant. We conclude that *atg6* overexpression has no effect in the absence of the core autophagy component *atg7*. The new data is shown in the new Suppl. Fig. S5 and the data presented in the Results section.

5. There are two paralogs of Atg8 in Drosophila: Atg8a that is expressed in all cells and Atg8b whose expression is practically restricted to the test is based on high-throughput data. Please specify

throughout the text and figures which Atg8 protein/gene is being looked at.

Response 3.5: All corrected.

6. A brief description of how trans-Tango labeling works would make it easier to understand those experiments.

Response 3.6: We have added a short explanation in the Results section as requested.

7. Fig S3. Only N-terminally tagged Atg8 reporters are functional as the C-terminus is cleaved by Atg4 to expose the Glycine residue that is conjugated to the lipid PE. Thus, please use GFP-Atg8(a?) instead of Atg8-GFP and specify which paralog (Atg8a vs Atg8a) it is. The same goes for Fig S7: Atg8-mCh should be corrected (mCherry-Atg8a?)

Response 3.7: We have applied these corrections as suggested

8. Line 268, "large Atg8-positive multivesicular bodies containing Syd-1" The true identity of the speculated multivesicular bodies is questionable - these may as well be autolysosomes. Please refrain from claiming this about the perinuclear Atg8a-positive vesicles, unless it is supported by eg. TEM.

Response 3.8: We have changed 'Atg8-positive multivesicular bodies' to 'Atg8-positive compartments'.

9. Line 332 what is "nenal"?

Response 3.9: 'neuronal', corrected with our apologies.

<u>Addendum: Figure for Reviewers</u>

The experiments in this Figure are described in <u>Response 1.4</u>.

We performed Lar antibody labeling in wild type brain with single CD4-tdGFP marked R7 axon terminals (green and middle column), where these GFP-marked axon terminals are either wild type (top row), mutant for lar (second row) or mutant for liprin-alpha (third row). In no case could we assign the punctate antibody labeling of all neurons to the marked axon terminals.

Similarly, the bottom two rows show the same experiment for Trio antibody labeling in wild type brains containing either wild type or trio mutant GFP-marked R7 axon terminals.

The antibodies and mutants were validated in independent experiments.

We conclude that levels of Lar and Trio in unmarked processes from other neurons within the marked regions (of which we know there are hundreds from EM reconstructions) 'dilute out' any quantifiable changes induced by mutating R7 axon terminals alone.

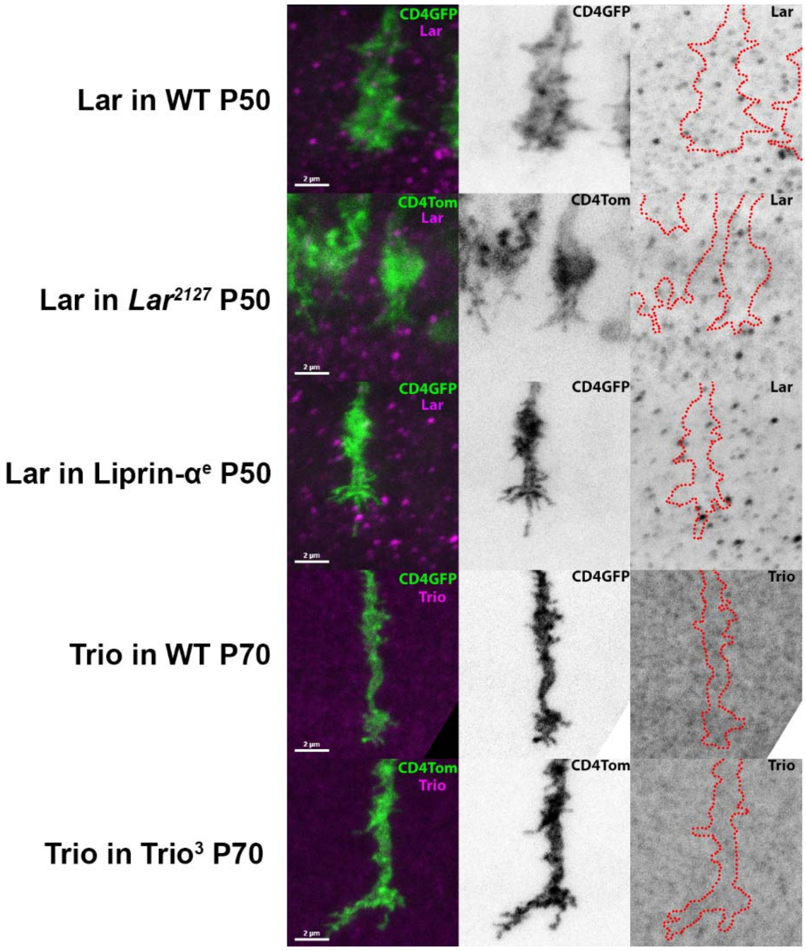

REVIEWERS' COMMENTS:

Reviewer #1 (Remarks to the Author):

In their revised manuscript and the accompanying rebuttal letter, the authors addressed the comments raised by the reviewers to the best possible extent. They took all suggestions into consideration, discussed each point separately, and performed additional experiments that strengthen their findings on the role of autophagy in synapse formation and synaptic partner choice during brain wiring in Drosophila melanogaster. They also adjusted text and figures accordingly. Thus the manuscript is now significantly improved. I only have some minor points that the authors might want to consider:

Fig. 2a-e’ (R7 terminals, lower panels).
Comparing to upper panel (R1 and R6) terminals, they seems to contain axons and soma. In case upper (R1 and R6) and lower (R7) panel terminals are not represented in the same way, please, indicate explicitly which parts are shown. Are the areas below red boxes proximal parts of R7 photoreceptors? Please, make clear that quantification in (g) represents whole R7 terminals and not only inside the red boxes. Have the authors included R8 photoreceptors in any experiment?

The authors suggest that autophagy mediates degradation of synaptogenic seeding factors. Indeed, they show that the autophagosomal marker Atg8 is co-localizes with Syd1, Liprin-a and Trio proteins. Do the levels of these proteins increase upon autophagy downregulation?

Line 95-96: The authors write: “atg18, (which) is required for PI3P recruitment to the phagophore and LC3 (Atg8) lipidation”. Actually, atg18 is recruited by PI3P to the phagophore and required for LC3 (Atg8) lipidation (the same mis-phrasing is found in response 3.2).

Line 211: Neither GFP-Atg5 nor GFP-LC3 marker (nor the combination) can track autophagosome maturation. Remove the word “maturation”.

Fig. 2h. Include color boxes indicating condition (grey: control, green: atg7, blue: atg6).

Supplementary Fig. 5a-b. Control (as Fig. 2a-a’) is missing.

Do Liprin-ɑ, Syd1 and/or Trio contain any (predicted) Atg8-interacting region motif?

Line 307: The suggested change wasn’t actually done. The word “multivesicular” is still in the text.

Table S2: f1: “cannot be measured”.

Reviewer #2 (Remarks to the Author):

The reviewers have successfully addressed my concerns by providing clear evidence that ATG5, ATG8a, Syd-1, or Liprin-alpha overexpression does not alter any of their readouts, demonstrated in new supplementary figures 7 & 12. The response to reviewers was thoughtful and a pleasure to read. Congratulations to the authors on an outstanding paper.

Reviewer #3 (Remarks to the Author):

During their revision, Kiral et al have addressed most of my concerns. I think that this work is now more convincing and it should be published after some minor corrections.

Minor comments:

1. Please specify Atg8a (based on the transgenes used) instead of the general term Atg8 in lines 1287-1291, 1399-1406, and in Fig S13c.

2. The authors state in their rebuttal that:
We have changed 'Atg8-positive multivesicular bodies' to 'Atg8-positive compartments'. (Response 3.8).
However, Line 307 still reads: (We) detected large Atg8-positive multivesicular bodies.
Please correct.

Responses to remaining questions by Reviewers 1 and 3

(original Reviewer comments *in full in blue* below):

Reviewer #1 (Remarks to the Author):

In their revised manuscript and the accompanying rebuttal letter, the authors addressed the comments raised by the reviewers to the best possible extent. They took all suggestions into consideration, discussed each point separately, and performed additional experiments that strengthen their findings on the role of autophagy in synapse formation and synaptic partner choice during brain wiring in Drosophila melanogaster. They also adjusted text and figures accordingly. Thus the manuscript is now significantly improved. I only have some minor points that the authors might want to consider:

Fig. 2a-e' (R7 terminals, lower panels).
Comparing to upper panel (R1 and R6) terminals, they seems to contain axons and soma. In case upper (R1 and R6) and lower (R7) panel terminals are not represented in the same way, please, indicate explicitly which parts are shown. Are the areas below red boxes proximal parts of R7 photoreceptors? Please, make clear that quantification in (g) represents whole R7 terminals and not only inside the red boxes. Have the authors included R8 photoreceptors in any experiment?

Figure Panels 2a-e' only show the synaptic regions of the axon terminals; for R1-R6 (top row) these are in the lamina, a non-layered neuropil, and for R7 (bottom row) the terminals are in the medulla, a layered neuropil. The terminals are represented in the same way, they just have somewhat different morphologies. We drew the red boxes only to highlight the region of R7 axon terminals that are normally almost devoid of synapses, but that do contain synapses in the autophagy mutants. Quantifications were done for all terminals in their entirety. To more clearly distinguish the different morphology of R7 terminals in the layer medulla neuropil, we have added the labeling of the medulla layers in the control panel 2a on the left. We updated and clarified the Figure legend.

We did not study photoreceptor R8 in any experiment in this paper.

The authors suggest that autophagy mediates degradation of synaptogenic seeding factors. Indeed, they show that the autophagosomal marker Atg8 is co-localizes with Syd1, Liprin-a and Trio proteins. Do the levels of these proteins increase upon autophagy downregulation?

To measure increased protein levels at filopodial tips we used immunohistochemistry. For quantification, we counted the filopodial tips containing synaptic seeding factors, as shown in Fig. 6i-k. The numbers of filopodia containing these proteins indeed increase upon autophagy downregulation. We know of no biochemical method to quantify the precise protein levels at the tips of dynamic filopodia inside the brain.

Line 95-96: The authors write: “atg18, (which) is required for PI3P recruitment to the

phagophore and LC3 (Atg8) lipidation". Actually, atg18 is recruited by PI3P to the phagophore and required for LC3 (Atg8) lipidation (the same mis-phrasing is found in response 3.2).

We apologize for this mistake and have corrected it according to the reviewer's suggestion.

Line 211: Neither GFP-Atg5 nor GFP-LC3 marker (nor the combination) can track autophagosome maturation. Remove the word "maturation".

We indeed only meant to describe the well-characterized progression from Atg5 to Atg8-positive autophagosomes. We have replace the word 'maturation' with 'progression'.

Fig. 2h. Include color boxes indicating condition (grey: control, green: atg7, blue: atg6).

We have included the legend in Fig. 2h.

Supplementary Fig. 5a-b. Control (as Fig. 2a-a') is missing.

We have added the control from Fig. 2.

Do Liprin-α, Syd1 and/or Trio contain any (predicted) Atg8-interacting region motif?

To our knowledge, nothing is known about direct interactions between these proteins. In the Discussion we raise the possibility that autophagy in the small space of a filopodial tip may in fact engulf cargo proteins unspecifically. The question of cargo-specificity in this process (as in so many autophagic processes) remains unresolved.

Line 307: The suggested change wasn't actually done. The word "multivesicular" is still in the text.

We apologize for the mistake. Now we changed it to "Atg8a-positive compartments".

Table S2: f1: "cannot be measured".

We have corrected this typo.

Reviewer #2 (Remarks to the Author):

The reviewers have successfully addressed my concerns by providing clear evidence that ATG5, ATG8a, Syd-1, or Liprin-alpha overexpression does not alter any of their readouts, demonstrated in new supplementary figures 7 & 12. The response to reviewers was thoughtful and a pleasure to read. Congratulations to the authors on an outstanding paper.

Reviewer #3 (Remarks to the Author):

During their revision, Kiral et al have addressed most of my concerns. I think that this work is

now more convincing and it should be published after some minor corrections.

Minor comments:

1. Please specify Atg8a (based on the transgenes used) instead of the general term Atg8 in lines 1287-1291, 1399-1406, and in Fig S13c.

We have corrected to Atg8a as advised.

2. The authors state in their rebuttal that:
We have changed 'Atg8-positive multivesicular bodies' to 'Atg8-positive compartments'. (Response 3.8).
However, Line 307 still reads: (We) detected large Atg8-positive multivesicular bodies. Please correct.

As noted in response to Reviewer 1, we apologize for the mistake. Now we changed it to "Atg8a-positive compartments".
